# Protein S-acylation controls the subcellular localization and biological activity of PHYTOCHROME KINASE SUBSTRATE

**DOI:** 10.1093/plcell/koad096

**Published:** 2023-03-28

**Authors:** Ana Lopez Vazquez, Laure Allenbach Petrolati, Martina Legris, Christophe Dessimoz, Edwin R Lampugnani, Natasha Glover, Christian Fankhauser

**Affiliations:** Centre for Integrative Genomics, Faculty of Biology and Medicine, University of Lausanne, Génopode Building, CH–1015 Lausanne, Switzerland; Centre for Integrative Genomics, Faculty of Biology and Medicine, University of Lausanne, Génopode Building, CH–1015 Lausanne, Switzerland; Centre for Integrative Genomics, Faculty of Biology and Medicine, University of Lausanne, Génopode Building, CH–1015 Lausanne, Switzerland; Department of Computational Biology, Faculty of Biology and Medicine, University of Lausanne, Génopode Building, CH–1015 Lausanne, Switzerland; Swiss Institute of Bioinformatics, University of Lausanne, Génopode Building, CH–1015 Lausanne, Switzerland; School of BioScience, University of Melbourne, Parkville, Victoria 3010, Australia; Menzies Institute for Medical Research, University of Tasmania, Hobart, Tasmania 7000, Australia; Department of Computational Biology, Faculty of Biology and Medicine, University of Lausanne, Génopode Building, CH–1015 Lausanne, Switzerland; Swiss Institute of Bioinformatics, University of Lausanne, Génopode Building, CH–1015 Lausanne, Switzerland; Centre for Integrative Genomics, Faculty of Biology and Medicine, University of Lausanne, Génopode Building, CH–1015 Lausanne, Switzerland

## Abstract

PHYTOCHROME KINASE SUBSTRATE (PKS) proteins are involved in light-modulated changes in growth orientation. They act downstream of phytochromes to control hypocotyl gravitropism in the light and act early in phototropin signaling. Despite their importance for plant development, little is known about their molecular mode of action, except that they belong to a protein complex comprising phototropins at the plasma membrane (PM). Identifying evolutionary conservation is one approach to revealing biologically important protein motifs. Here, we show that PKS sequences are restricted to seed plants and that these proteins share 6 motifs (A to F from the N to the C terminus). Motifs A and D are also present in BIG GRAIN, while the remaining 4 are specific to PKSs. We provide evidence that motif C is S-acylated on highly conserved cysteines, which mediates the association of PKS proteins with the PM. Motif C is also required for PKS4-mediated phototropism and light-regulated hypocotyl gravitropism. Finally, our data suggest that the mode of PKS4 association with the PM is important for its biological activity. Our work, therefore, identifies conserved cysteines contributing to PM association of PKS proteins and strongly suggests that this is their site of action to modulate environmentally regulated organ positioning.

## Teaser

Members of the PHYTOCHROME KINASE SUBSTRATE (PKS) family are characterized by six conserved sequence motifs, one of which is required for plasma-membrane association and biological activity of PKS4.

**IN A NUTSHELL**

**Background:** Plants can orient their leaves towards the light. This is known as phototropism and fascinated scientists since Charles Darwin. Phototropism is initiated by a blue light photoreceptor called phototropin. Light activates phototropin's protein kinase activity (an enzyme that phosphorylates proteins). This is followed by a series of poorly understood events, which lead to the redistribution of the growth hormone auxin in the stem resulting in growth towards the light. PHYTOCHROME KINASE SUBSTRATE 4 (PKS4) is phosphorylated by phototropin and is believed to act between phototropin light activation and auxin redistribution. The mechanism of PKS4 action is unknown because the protein does not contain any domains of known biochemical activity. PKS4 associates with the plasma membrane (PM), where it was postulated to act.

**Question:** We wanted to understand how PKS4 associates with the PM and determine whether this subcellular localization is important for its biological activity.

**Findings:** Functionally important parts of proteins are typically conserved over evolutionary timescales. We, therefore, identified genes coding for PKS proteins and found that they are present in seed plants and comprise 6 conserved sequence motifs. We showed that one of these motifs is important for efficient PKS association with the PM in Arabidopsis. Cysteine residues of this motif are modified with lipids, which presumably contribute to PM association. Moreover, in PKS4, these residues are important for its function in phototropin signaling in Arabidopsis.

**Next steps:** Our study identified a PKS4 sequence motif that is important for biological activity and subcellular localization. However, we still don’t know what the rest of the protein does and how it may be important in linking phototropin activation and auxin redistribution. We hope that studying other conserved motives identified here will allow us to answer this question.

## Introduction

The direction of hypocotyl growth is part of a developmental program that begins with early seedling establishment that influences the physiology and development of adult plants (Galen et al. 2004). In Arabidopsis (*Arabidopsis thaliana*), etiolated (dark-grown) seedlings grow following the direction of the constantly present gravity stimulus: roots grow downwards while shoots grow upwards. However, in response to light, hypocotyls orient their growth following the integration of 2 main signaling pathways: gravitropism (repressed) and phototropism (activated) (Correll and Kiss 2002). Growth against the gravity vector of etiolated hypocotyls is inhibited by the perception of red light (RL) and far-red light (FRL), leading to a random orientation of hypocotyl growth, which is referred to as inhibition of gravitropism and involves the RL and FRL photoreceptors phytochromes (phy) (Robson and Smith 1996). Additionally, plants perceive the directionality of blue light (BL) to orient their photosynthetic organs towards the light source to increase light capture, which is known as phototropism. Despite the influence of phytochromes and cryptochromes, phototropism is mainly controlled by the BL photoreceptors phototropins (phot) (Goyal et al. 2013). Although phototropism and inhibition of gravitropism are independent responses, phy-mediated inhibition of gravitropism has been proposed to enhance phototropism in response to BL (Lariguet and Fankhauser 2004; Liscum et al. 2014).

Gravitropism signaling comprises the perception of the gravity vector, which requires sedimentation of starch-filled amyloplasts for signal generation and transduction, resulting in hypocotyls growing upwards (Sack 1997; Nakamura et al. 2019; Vandenbrink and Kiss 2019). In Arabidopsis, basic helix-loop-helix transcription factors of the PIF (PHYTOCHROME-INTERACTING FACTOR) family regulate gravitropism in darkness (Oh et al. 2004). In response to RL and FRL, phytochromes convert hypocotyl amyloplasts into plastids with chloroplast properties that show a lower starch composition, which diminishes the ability of seedlings to sense gravity; consequently, hypocotyls show a randomized growth orientation (Kim et al. 2011). PIFs inhibit the conversion of amyloplasts to other plastids in the dark; however, RL-mediated phyB activation in the epidermis promotes the degradation of endodermal PIFs, which releases PIF-imposed inhibition of amyloplast conversion (Kim et al. 2016a, 2016b). In addition to the phy-PIF module, the phytochrome-interacting PKS (PHYTOCHROME KINASE SUBSTRATE) protein family acts in phytochrome signaling to regulate RL- and FRL-mediated growth responses (Fankhauser et al. 1999; Lariguet et al. 2003). PKS1 and more prominently PKS4 regulate the inhibition of gravitropism in response to RL and FRL (Schepens et al. 2008). However, the mechanism behind the function of PKS proteins remains unknown.

Arabidopsis has 2 phototropins, which exhibit specific and partially redundant functions: phot1 and phot2. Hypocotyl phototropism is mainly mediated by phot1 (Christie 2007). Phototropins form a protein complex with members of the NRL (NONPHOTOTROPIC HYPOCOTYL 3 [NPH3] and ROOT PHOTOTROPISM2-LIKE [RPT2-LIKE]) and PKS families, which are involved in the early steps of phot-mediated signaling (Christie et al. 2018; Harmer and Brooks 2018). Phototropins are serine/threonine (Ser/Thr) protein kinases belonging to the AGC family (cAMP-DEPENDENT PROTEIN KINASE, cGMP-DEPENDENT PROTEIN KINASE G, and PHOSPHOLIPID-DEPENDENT PROTEIN KINASE C) that phosphorylate NPH3 and PKS4 in response to BL (Demarsy et al. 2012; Christie et al. 2018; Schumacher et al. 2018; Sullivan et al. 2021). Despite their hydrophilic properties, phototropins associate with the plasma membrane (PM) where they initiate the light signaling cascade (Preuten et al. 2015). BL leads to phot1 homodimerization, phosphorylation, and translocation to functional membrane microdomains where signal transduction is activated (Xue et al. 2018). Phototropic curvature is initiated by a higher activation of phot1 on the irradiated relative to the shaded side of the hypocotyl. This difference leads to asymmetric NPH3 aggregation, correlating with a phot1-activation gradient (Sullivan et al. 2019; Legris and Boccaccini 2020). However, how this phot1-activation gradient across the hypocotyl leads to an auxin gradient finally resulting in growth reorientation remains poorly understood (Fankhauser and Christie 2015).

PKSs are a family of basic hydrophilic proteins that do not contain domain(s) of known function. They were initially identified as phytochrome-binding proteins that regulate phytochrome signaling (Fankhauser et al. 1999). Surprisingly, despite their hydrophilic nature, they are associated with the PM (Lariguet et al. 2006; de Carbonnel et al. 2010; Demarsy et al. 2012). *PKS*s are expressed in the hypocotyl elongation zone, consistent with their importance during hypocotyl growth regulation (Lariguet et al. 2003; Schepens et al. 2008; Kami et al. 2014). PKS1, PKS2, and PKS4 form a protein complex with phot1 and NPH3 possibly mediating the link between phot1 activation and auxin gradient formation, which ultimately leads to hypocotyl growth towards the light (Lariguet et al. 2006; de Carbonnel et al. 2010; Kami et al. 2014; Schumacher et al. 2018). However, the molecular mode of action of PKSs remains unknown, which prompted us to conduct a structure-function study of PKS proteins. Although our phylogenetic analyses revealed a low overall similarity among PKS members within seed plants, we identified 6 short regions of protein similarity that we called motifs A to F. We identified motif C as a key determinant of PKS subcellular localization. Further characterization showed that conserved cysteines in this motif are S-acylated and are required for membrane localization and PKS4 function.

## Results

### Phylogeny and motif organization of PKS proteins

**Figure 1.**
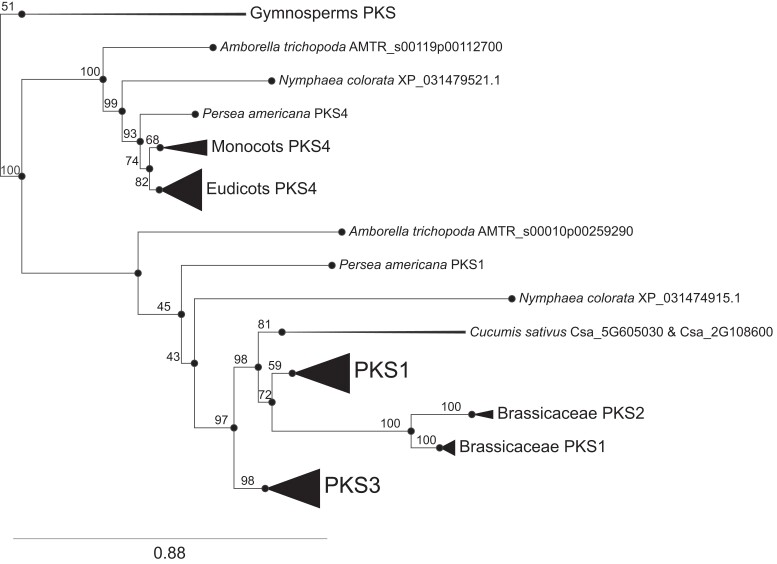
PKS proteins are present in seed plants. Simplified phylogeny of PKS proteins, obtained using IQ-TREE and the JTT + I + G4 substitution model. Some nodes are collapsed, as indicated by triangles at the leaves and represent multiple proteins. Ultrafast bootstrap values are shown at each node, where available. The full version of the tree can be found in Supplemental Fig. S2.

Using the D^2^P^2^ sequence predictor (Oates et al. 2012), we determined that the primary amino-acid sequence of Arabidopsis PKS proteins is predicted to be largely disordered and lacks any domain of known function (Supplemental Fig. S1). To define functionally important regions of PKS proteins, we used a phylogenetic approach to identify evolutionarily conserved sequences. To this end, we identified 172 *PKS* homologs using the Orthologous matrix (OMA) browser (Altenhoff et al. 2021) and manual reciprocal BLASTp searches in NCBI and dedicated plant genome databases (Supplemental Data Set 1). This analysis revealed the presence of *PKS* genes in all angiosperms and a few sequences in gymnosperms (Fig. 1). However, our searches did not reveal related sequences in other orders of land plants such as in ferns, mosses, or the liverwort *Marchantia polymorpha*. We determined the phylogeny by aligning the 172 PKS protein sequences identified above using MAFFT (Katoh and Standley 2013), removing columns with gaps in more than 20% of the sequences using trimAl (Capella-Gutierrez et al. 2009), and then building a maximum-likelihood tree using IQ-TREE and 1,000 ultrafast bootstrap replicates (Trifinopoulos et al. 2016; Hoang et al. 2018). The resulting phylogenetic tree had a number of internodes with ultrafast bootstrap support values ≤95, which are considered unreliable (Minh et al. 2013). However, given the reliable support values we obtained at other branches, we detected a clear distinction between PKS4 and PKS1/PKS3, PKS1 and PKS3, and Brassicaceae PKS1 and PKS2 (Fig. 1). In the basal angiosperms *Amborella trichopoda*, *Nymphaea colorata*, and avocado (*Persea americana*), we identified 2 *PKS* genes each, which group into the PKS4 subfamily and a subfamily formed by the ancestors of PKS1, PKS2, and PKS3 (thereafter referred to as the PKS1/2/3 subfamily, with names based on the Arabidopsis genes). Based on the limited data available from gymnosperms and the limited bootstrap support throughout the tree, the most parsimonious interpretation of our data is that *PKS* was present as a single copy in the ancestral spermatophyte genome (seed plants, including gymnosperms and angiosperms). We hypothesize that there was a duplication in the ancestral angiosperm to form 2 copies: *PKS4* and *PKS1/2/3*. Another duplication occurred after the divergence of *Amborella*, *P. americana*, and *N. colorata*, giving rise to *PKS1/2* and *PKS3* in the ancestral Mesangiospermae. An additional Brassicaceae-specific duplication gave rise to *PKS1* and *PKS2*. *PKS4* genes are present in all analyzed monocot and dicot genomes (Fig. 1A and Supplemental Fig. S2). Eudicots typically possess additional members of the *PKS1/2/3* subfamily, while in Brassicaceae the *PKS1* subfamily further duplicated into *PKS1* and *PKS2*. In addition to *PKS4*, monocot genomes often also contain additional *PKS* genes presumably belonging to the *PKS1/2/3* subfamily. These sequences are not represented here because they are more difficult to identify due to greater sequence divergence. We conclude that *PKS* sequences are present in seed plants, basal angiosperms possess *PKS* genes from 2 subfamilies, and in eudicots, the gene family further duplicated leading to the presence of *PKS* sequences from at least 3 subfamilies (*PKS4*, *PKS3*, and *PKS1*).

**Figure 2.**
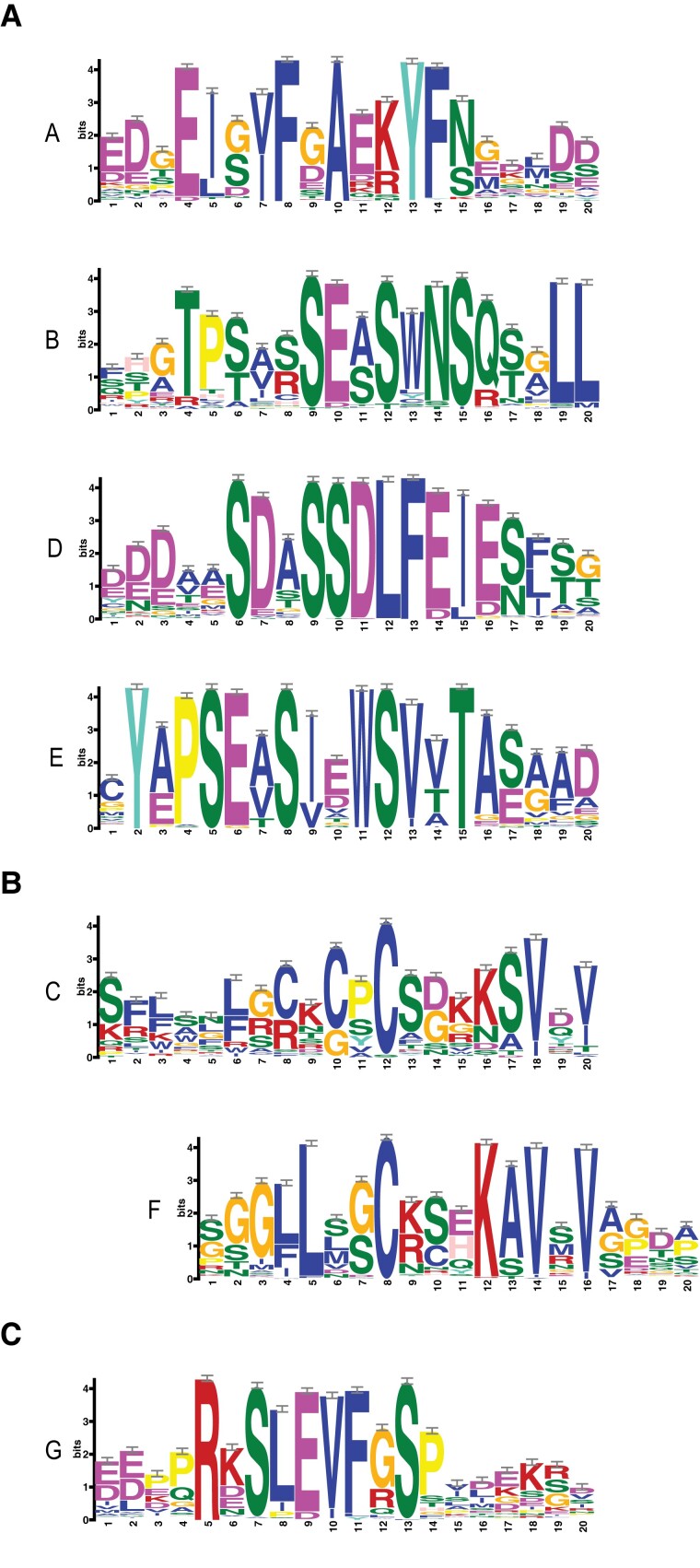
Protein motifs characterizing PKS proteins. **A)** Sequence motifs conserved among members of the PKS family. Motifs A to F are found from the N to the C terminus of the proteins. GLAM2 was used to find the conserved motifs and to create the logo images: Motifs A, B, D, and E. **B)** Motifs C and F are related to each other downstream of the central Cys residue. **C)** Conserved motif G, which is absent in Brassicaceae PKS3 and PKS4 proteins.

To define conserved sequence motifs, we used 172 taxonomically divergent PKS protein sequences from all 4 subfamilies (Supplemental Table S1) as input for GLAM2 (Frith et al. 2008). We established that these proteins are characterized by the presence of 6 conserved motifs that we call A to F (from the N to the C terminus) (Fig. 2). Although the order of motifs is fully conserved among PKS proteins, the degree of sequence conservation and the spacing between each motif is much more variable. In addition, a comparison of motifs revealed significant similarity (*P*<0.05) between motifs C and F, suggesting that parts of these motifs are related to each other (Fig. 2B). Finally, many PKS proteins comprise an additional conserved motif that we call G, which is present between motifs C and D (Fig. 2C). Given that this sequence motif is absent from Brassicaceae PKS3 and PKS4 (including Arabidopsis) (Supplemental Fig. S2), we did not consider this motif further.

**Figure 3.**
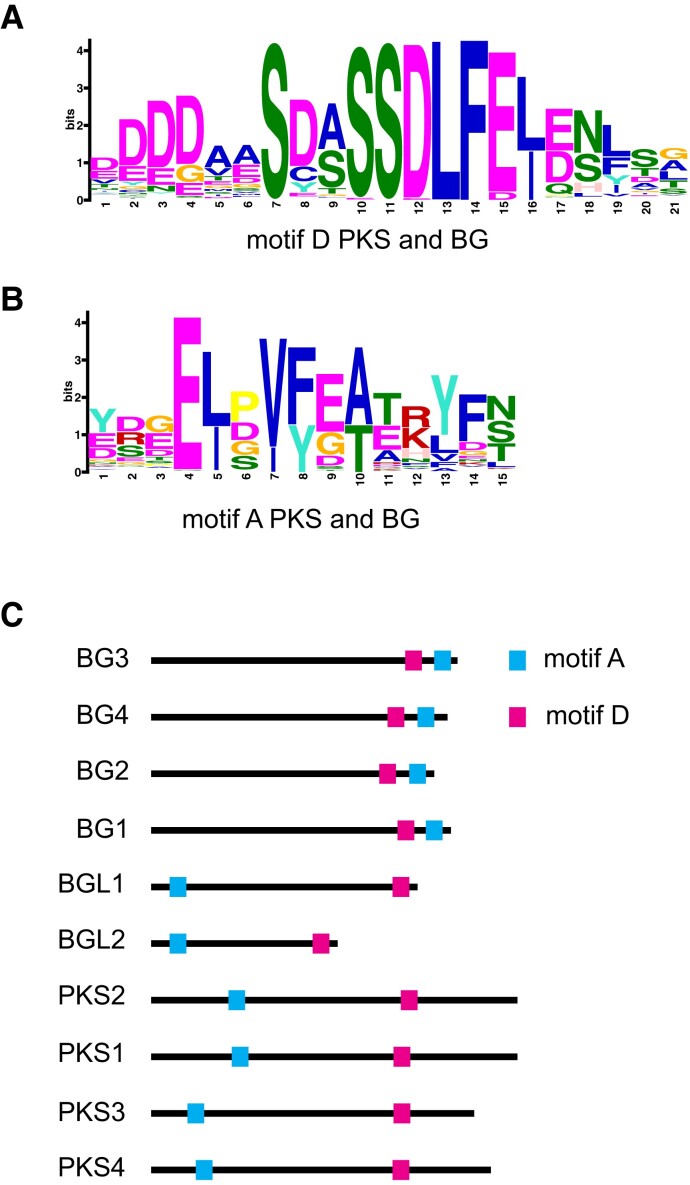
PKS and BIG GRAIN proteins share common sequence motifs. **A)** Sequence motif D is conserved among members of the PKS and BIG GRAIN (BG) families. **B)** Sequence motif A is conserved among members of the PKS and BG families. **C)** Protein motif organization of *A. thaliana* PKS, BG, and BG-LIKE (BGL) proteins. The proteins are drawn to scale; motif A, blue box; motif D, pink box.

We used hidden Markov model-based protein searches (Gabler et al. 2020) to determine whether these motifs are present in other proteins: indeed, we detected a conserved sequence related to motif D towards the C terminus of BIG GRAIN (BG and BG-LIKE) proteins (Mishra et al. 2017). To investigate this relationship in more detail, we combined our list of PKS sequences (172 proteins, Supplemental Data Set 1) and BG proteins (219 proteins) retrieved from the OMA database (Altenhoff et al. 2021). We used all these sequences to find motifs common to both BG and PKS proteins. A MEME search (Bailey and Elkan 1994) showed that 390/391 of these sequences share motif D and 379/391 share a motif similar to A (Fig. 3). Interestingly, the order of these motifs is not the same in all members of the BG family, as illustrated by the Arabidopsis proteins. While in Arabidopsis BG-LIKE proteins motif A precedes motif D as in PKS proteins, in BG proteins motif A is immediately downstream of motif D (Fig. 3C). This analysis revealed that PKS, BG, and BG-LIKE proteins are related and have a similar taxonomic distribution (Figs. 1 to 3) (Mishra et al. 2017). Unfortunately, the molecular function of these conserved motifs is presently unknown (Mishra et al. 2017). In summary, our sequence analyses identified 6 conserved motifs present in PKS proteins, and we hypothesize that they correspond to functionally important portions of these proteins.

### Motif C of PKS proteins is S-acylated and required for their association with the PM

**Figure 4.**
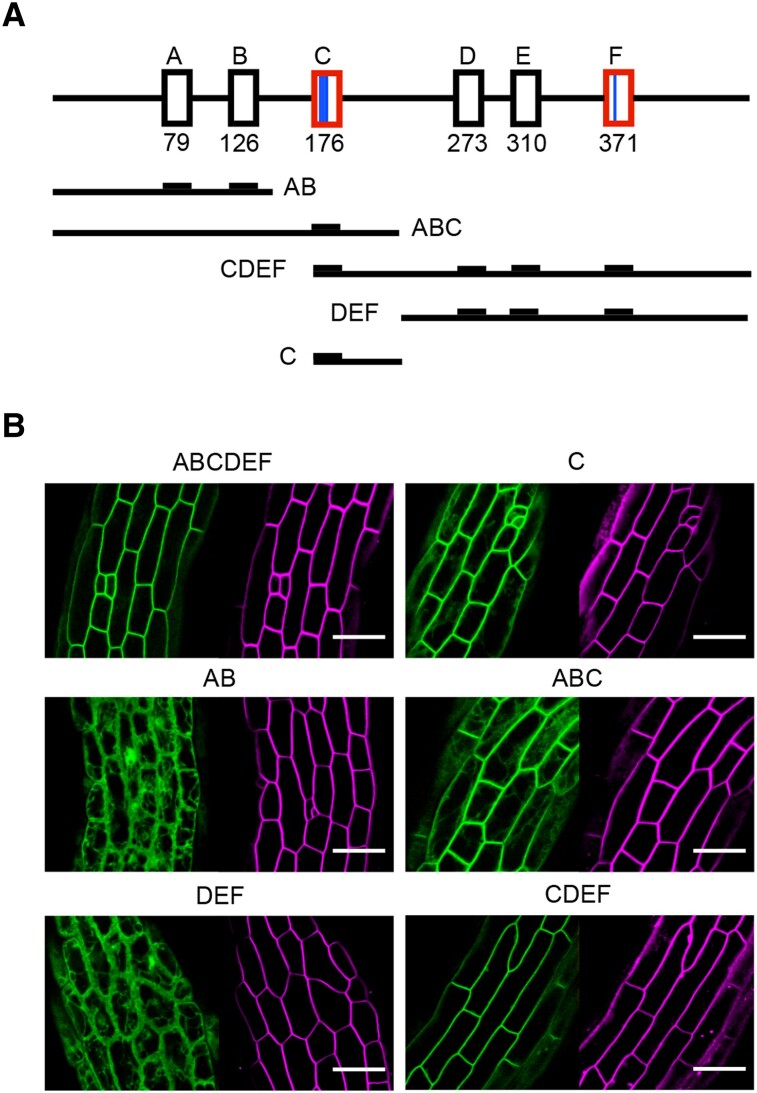
PKS1 is PM-associated and motif C is required for this association. **A)** Illustration of PKS1 protein (and PKS1 truncations shown in **B**) with the 6 motifs as defined in Fig. 2. The number under each box corresponds to the starting amino acid in the Arabidopsis PKS1 sequence. Motifs C and F are shown in red, as they share homology. Cys residues are shown as blue lines; 1 thick line in motif C to represent the 3 Cys residues, 1 thin line in motif F to represent 1 cysteine residue. **B)** Confocal images of epidermal hypocotyl cells from 3-d-old transgenic etiolated seedlings, accumulating GFP-tagged PKS1 either as full-length or truncated fragments. FM4-64 staining (magenta) shows the PM. Scale bars, 30 *µ*m.

PM localization is presumably a functionally relevant feature of PKS proteins (Lariguet et al. 2006; de Carbonnel et al. 2010; Schumacher et al. 2018). To test whether one of the conserved sequence motifs controls PKS1 subcellular localization, we examined the localization of a series of PKS1 truncations fused to the green fluorescent protein (GFP) in hypocotyl epidermal cells of stably transformed Arabidopsis seedlings (Fig. 4A). The *PKS1-GFP* sequence was placed under the control of the cauliflower mosaic virus (CaMV) 35 promoter. We first confirmed that the full-length (ABCDEF) PKS1-GFP signal was consistent with the previously reported PM localization (Fig. 4). We observed that both ABC-GFP and CDEF-GFP colocalize with the dye FM4-64, in agreement with PM localization (Fig. 4B). By contrast, AB-GFP and DEF-GFP truncated proteins largely lost PM localization, suggesting that motif C plays a central role in mediating the association of PKS1 to the PM. Consistent with this idea, a portion of PKS1 consisting of conserved motif C alone was sufficient for localizing a GFP fusion protein to the PM (Fig. 4B).

**Figure 5.**
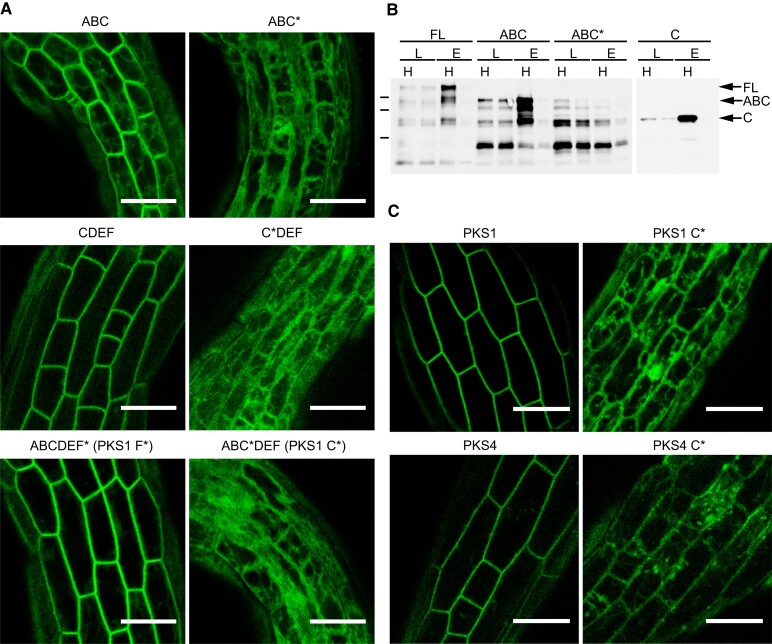
Cysteines of motif C from PKS1 and PKS4 are essential for PM localization. **A)** Confocal images of epidermal hypocotyl cells from 3-d-old transgenic etiolated seedlings, accumulating GFP-tagged PKS1 full-length (FL) or truncated fragments with mutated cysteines in either motif C or motif F. Scale bars, 30 *µ*m. **B)** Cysteine residues of motif C are S-acylated. Immunoblots of FL PKS1 or truncated fragments either with the intact sequence (ABC) or with mutated cysteines (ABC*). Protein extracts were used in a biotin switch assay. L, loading; E, elution; H, hydroxylamine-treated. Marker sizes are 72, 55, and 36 kD. **C)** Confocal images of epidermal hypocotyl cells from 3-d-old transgenic etiolated seedlings, accumulating GFP-tagged FL PKS1 or PKS4 proteins, either intact (WT) or harboring Cys-to-Ser mutations in motif C. Scale bars, 30 *µ*m.

Highly conserved Cystine (Cys) residues (invariant Cys at position 12, and less conserved Cys at positions 10 and 8; Fig. 2) are a striking feature of motif C. Such residues can be acylated to mediate PM association (Hemsley 2020). To assess their importance in controlling PKS1 subcellular localization, we mutated all 3 Cys-to-Ser residues, which cannot be acylated. Mutating these residues in the context of the *ABC-GFP*, *CDEF-GFP*, or full-length *PKS1-GFP* constructs strongly altered the ability of the encoded proteins to associate with the PM (Fig. 5A). To test if the loss of association with the PM was specific to the conserved Cys residues in motif C, we also mutated the conserved Cys residue in motif F to Ser. Importantly, this mutation did not alter the subcellular localization of PKS1-GFP (Fig. 5A). This result is consistent with our observation that CDEF-GFP but not DEF-GFP associates with the PM, indicating the central role of motif C in targeting the protein to the PM, which is apparently mediated by conserved Cys residues (Fig. 4B). To determine whether these conserved Cys residues controlling the PM association of PKS1 are S-acylated, we performed a biochemical assay with the same transgenic plants used for microscopy analysis. The assay consists of exchanging acyl groups covalently bound to Cys residues with biotin, which is then detected by affinity purification (Hemsley 2013). This assay showed that full-length PKS1-GFP and ABC-GFP are both acylated, while mutating the conserved Cys residues in the latter protein prevented acylation. Moreover, the C-GFP fusion protein was acylated as well, which is consistent with acylation of at least one of the conserved residues of motif C in PKS1. To investigate whether the conserved Cys residues in motif C are also important to control the subcellular localization of other PKS proteins, we selected PKS4, a member of the other major PKS subfamily (Fig. 1). While PKS1 proteins typically have 3 Cys residues in motif C, the most highly conserved one at position 12, a second one at position 10, and a third at position 8, PKS4 proteins typically have 2: Cys 12 and Cys 10 (Fig. 2; Supplemental Fig. S3). We thus substituted both Cys residues with Ser in the context of full-length *PKS4-GFP* driven by the 35S promoter and generated stable transgenic Arabidopsis plants in the Col-0 background. While as reported previously (Schumacher et al. 2018), PKS4-GFP was associated with the outline of the cell (Fig. 5C), mutations of the conserved Cys residues in motif C strongly impaired this PM association, as with PKS1 (Fig. 5C). Collectively, these results indicate that conserved cysteines in motif C are acylated and essential to mediate the PM association of PKS1 and PKS4.

### The conserved cysteines in motif C of PKS4 are required for biological activity

**Figure 6.**
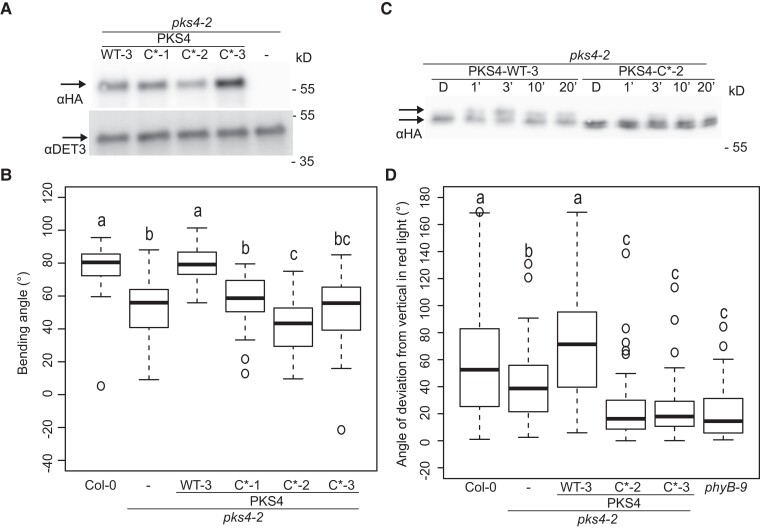
Motif C is required for PKS4 function in phototropism and inhibition of gravitropism. **A)** Immunoblot analysis of PKS4 tagged with an HA tag, using an anti-HA antibody on total protein samples extracted from *pks4-2*, *pks4-2 PKS4* WT-3 and pks4-2 PKS4 C*-1, pks4-2 PKS4 C*-2, and pks4-2 PKS4 C*-3 samples of 3-d-old dark-grown seedlings. The same membrane was probed with anti-DET3 antibodies as a loading control. **B)** Phototropic curvature of 3-d-old dark-grown Col-0, *pks4-2*, *pks4-2 PKS4* WT-3, and *pks4-2 PKS4* C* (C*-1, C*-2, and C*-3) lines treated with unidirectional BL. Seedlings were exposed to 0.1 *µ*mol m^−2^ s^−1^ BL for 24 h prior to measurement of growth reorientation. *n* = 40 to 60, means with the same letter are not significantly different (*P* > 0.01, one-way ANOVA with Tukey's HSD test). **C)** Immunoblot analysis of HA-tagged PKS4 using an anti-HA antibody on total protein samples extracted from *pks4-2 PKS4* WT and pks4-2 PKS4 C*-2 of 3-d-old dark-grown seedlings exposed to 1 *µ*mol m^−2^ s^−1^ BL for 1, 3, 10, or 20 min. **D)** Hypocotyl growth orientation of Col-0, *pks4-2*, *pks4-2 PKS4* WT-3 and pks4-2 PKS4 C*-1, C*-2, and C*-3 seedlings growing in continuous RL (30 *µ*mol m^−2^ s^−1^). Seedlings were kept in darkness for 24 h prior to 4 d of RL treatment, after which growth orientation was measured. 0° represents vertical growth. We consider the absolute value of the angle, whether the seedling bends towards the left or the right side. *n* = 70 to 80, different lowercase letters are significantly different (*P* > 0.01, one-way ANOVA with Tukey's HSD test).

To determine whether the conserved Cys residues in motif C are functionally relevant, we attempted to complement *pks4*, which is defective in phototropism and phytochrome-mediated inhibition of hypocotyl gravitropism (Schepens et al. 2008; Kami et al. 2014). We chose the *pks4* single mutant, as it displays the strongest defects in phototropism and gravitropism among all *pks* single mutants (Schepens et al. 2008; Kami et al. 2014). We used a construct encoding the same PKS4 motif C variant as for the subcellular localization studies above (Fig. 5C) but with a PKS4 carboxyl-terminal triple HA tag; we placed the entire coding sequence under the control of a 1.5-kb *PKS4* promoter fragment. We generated transgenic *pks4* plants expressing this *PKS4* variant in motif C (termed PKS4 C* lines) and selected 3 independent single insertion lines accumulating PKS4 to levels comparable to a wild-type PKS4-HA control line (WT-3) (Fig. 6A) (Schumacher et al. 2018). The *PKS4* WT-3 line showed complementation of the phototropic phenotype of the *pks4* mutant as previously shown (Fig. 6B) (Schumacher et al. 2018). However, none of the 3 independent PKS4 C* lines complemented the mutant (Fig. 6B). Additionally, we observed that one of these lines (PKS4 C*-2) exhibits an even stronger phototropic defect than the *pks4* mutant, suggesting that the expression of the PKS4 C* variant might interfere with the molecular mechanism underlying phototropism. To look at the rapid phototropin-mediated response, we analyzed the light-induced reduction of PKS4 mobility on SDS-PAGE gels triggered by phototropin-mediated PKS4 phosphorylation (Demarsy et al. 2012). PKS4 C* from etiolated seedlings had a somewhat different migration pattern than PKS4, migrating as 2 bands. This difference may be due to the slightly altered amino-acid composition of the mutant and/or a change in protein acylation. In response to BL, we observed the previously described gradual and transient increase in the appearance of a slower migrating PKS4 isoform in the *PKS4* WT line (Fig. 6C) (Demarsy et al. 2012). This pattern was largely abolished in the PKS4 C*-2 line, in which the BL-induced slower migrating isoform of PKS4 is very faint (Fig. 6C). We observed the same difference in migration between the *PKS4* WT and the PKS4 C*-1 line (Supplemental Fig. S4A). Collectively, our results show that mutating conserved Cys residues in motif C impairs the ability of PKS4 to promote phototropism and appears to limit the ability of phot1 to phosphorylate PKS4.

To determine whether motif C is also important for the function of PKS4 in phytochrome signaling, we characterized light-induced inhibition of hypocotyl gravitropism (Schepens et al. 2008) by determining hypocotyl growth orientation (relative to the vertical) in seedlings grown in continuous RL. We found that the control PKS4 WT-3 line rescued this light response, but none of the PKS4 C* lines complemented *pks4* (Fig. 6D). The phenotype of these lines was more pronounced than the *pks4* phenotype, resembling that seen in the *phyB* mutant (Fig. 6D). Therefore, PKS4 C* enhances the *pks4* null phenotype, suggesting that it interferes with PKS function during phytochrome-mediated inhibition of hypocotyl gravitropism. We also analyzed hypocotyl gravitropism in darkness and found that the WT Col-0, *pks4*, and all transgenic lines show the same response (Supplemental Fig. S4B) confirming that the effect of PKS4 on hypocotyl gravitropism is light-dependent. Collectively our data indicate that the highly conserved Cys residues of motif C are essential for the function of PKS4 in phytochrome and phototropin signaling.

**Figure 7.**
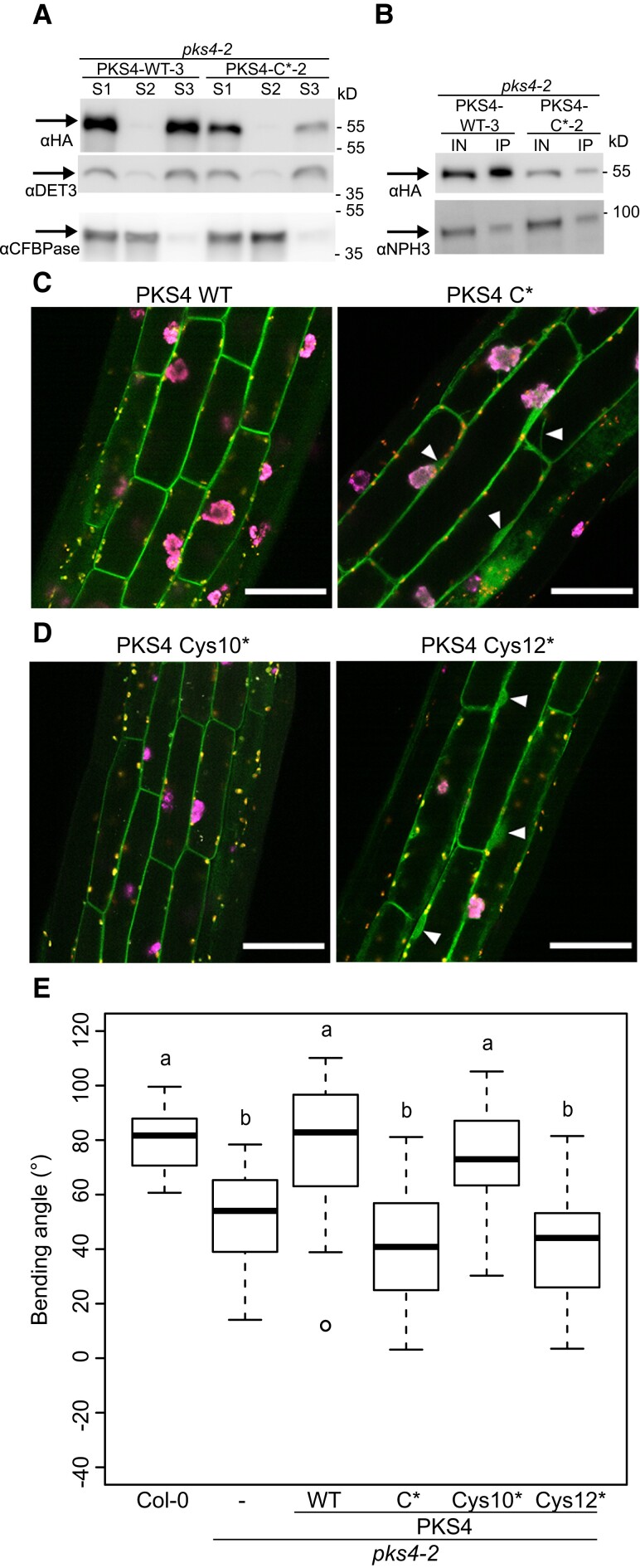
Characterization of the PKS4 motif C mutant. **A)** Cellular fractionation of wild-type PKS4 and the PKS4 C* mutant from etiolated seedlings. S1 corresponds to total proteins, S2 to the cytosolic fraction, and S3 the microsome fraction. Proteins from these fractions were separated by SDS-PAGE transferred onto a membrane that was probed with anti-HA to detect PKS4, anti-DET3 as a marker of cell membranes, and anti-CFBPase as a cytosolic marker. **B)** PKS4 and PKS4 C* co-immunoprecipitate with NPH3. Solubilized microsome fractions prepared from etiolated seedlings harboring the *PKS4* WT and mutant PKS4 C*-HA constructs were immunoprecipitated with an anti-HA antibody. The input (IN) and immunoprecipitate (IP) were separated by SDS-PAGE before being transferred onto a membrane that was probed with anti-HA to detect PKS4 and with anti-NPH3 antibodies. **C)** and **D)** Localization of PKS4 variants, tagged with GFP and encoded by a construct driven by the *PKS4* promoter, in hypocotyl cortex cells of 3-d-old etiolated seedlings. Scale bars, 50 *µ*m. White arrowheads mark some of the intracellular GFP signal present in C* (Cys-10 and Cys-12 mutated to Ser) and Cys12* variants. **E)** Phototropic curvature of 3-d-old dark-grown Col-0, *pks4-2*, *pks4-2 PKS4* WT, pks4-2 PKS4 C*, pks4-2 PKS4 Cys-10*, and pks4-2 PKS4 Cys-12* seedlings treated with unidirectional BL. Primary transformants in the *pks4-*2 background expressing *PKS4* WT and the different variants were assayed. Seedlings were exposed to 0.1 *µ*mol m^−2^ s^−1^ BL for 24 h prior to measurement of growth reorientation. *n* = 30 to 50, different lowercase letters are significantly different (*P* > 0.01, one-way ANOVA with Tukey's HSD test).

Our results established that the PKS4 C* variant shows impaired localization to the PM (Fig. 5C), exhibits less light-induced phosphorylation than full-length PKS4, and fails to complement the phenotypes of *pks4* seedlings (Fig. 6). To further characterize the C* mutant, we fractionated cell extracts from etiolated seedlings using the HA-tagged lines accumulating WT levels of the proteins encoded by each transgene (Fig. 6). This experiment revealed that both PKS4 C* and intact PKS4 are enriched in the microsome fraction (Fig. 7A) (Demarsy et al. 2012). We then solubilized these microsomes, immunoprecipitated PKS4 (WT and C* variant), and observed that both PKS4 WT and PKS4 C* interact with NPH3, as seen by a co-immunoprecipitation with anti-HA antibodies, followed by immunoblotting with an anti-NPH3 antibody (Fig. 7B). As we performed our initial microscopy analysis of PKS4 in epidermal cells of seedlings overexpressing *PKS4* from the 35S promoter (Fig. 5C), we decided to determine the subcellular localization in seedlings expressing *PKS4* from its own promoter. These studies showed that PKS4 most strongly accumulates in hypocotyl cortex cells, where we detected PKS4 mostly associated with the cellular periphery in *PKS4:PKS4-GFP* lines, consistent with a PM association (Fig. 7C). By contrast, we consistently observed more intracellular signal in PKS4pro:PKS4 C*-*GFP* lines indicative of a reduced ability for the C* mutant protein to localize to the PM (Fig. 7C, and Supplemental Fig. S4D shows additional independent lines). Motif C of PKS4 proteins contains 2 Cys residues corresponding to the most conserved Cys-12 and Cys-10 (Fig. 2B and Supplemental Fig. S3A). We, therefore, also generated constructs expressing the sequences encoding a PKS4 Cys-10 or a Cys-12 mutant (both to Ser) fused to GFP from the *PKS4* promoter. We introduced these constructs into the *pks4* mutant background. Microscopy examination of hypocotyl cortex cells showed that while the Cys-10-Ser mutant shows a subcellular localization pattern comparable to the wild type, the Cys-12-Ser mutant behaved very similarly to the PKS4 C* mutant (with both Cys-10 and Cys-12 being mutated) (Fig. 7, C and D, and Supplemental Fig. S4E for independent Cys-10 and Cys-12 mutant lines). This result indicates that the fully conserved Cys-12 residue is critical for PKS4 subcellular localization. We introduced similar constructs with an HA tag instead of GFP into the *pks4* mutant and used them for complementation assays in the T1 generation. Using T1 plants allows for the comparison of dozens of independent transgenic events and, therefore, mitigates for position effects of the insertion. This experiment indicated that while the Cys-10 mutant can complement the phototropic defect of *pks4*, the Cys-12 mutant, like the PKS4 C* variant, was unable to do so (Fig. 7E). We conclude that the ability of PKS4 to associate with the PM appears to correlate with its complementation potential.

**Figure 8.**
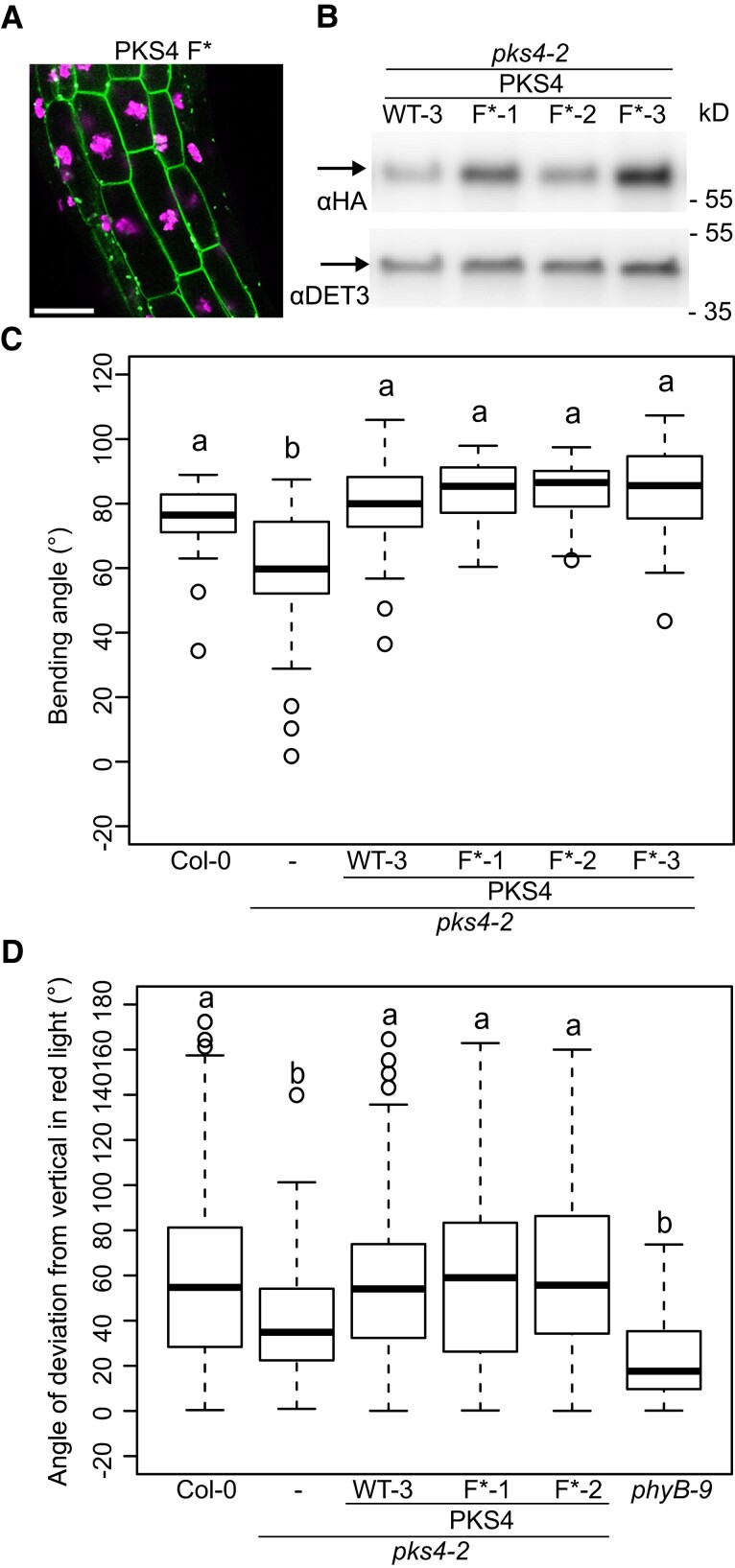
The invariant Cys residue of motif F is not required for PKS4 function. **A)** Localization of the PKS4 F* variant, tagged with GFP and encoded by a construct driven by the *PKS4* promoter in hypocotyl cortex cells of 3-d-old etiolated seedlings. Scale bar, 50 *µ*m. **B)** Immunoblot analysis of HA-tagged PKS4 using an anti-HA antibody on total protein samples extracted from *pks4-2 PKS4* WT-3 and pks4-2 PKS4 F*-1, F*-2, and F*-3 3-d-old dark-grown seedlings. The same membrane was probed with anti-DET3 antibodies as a loading control. **C)** Phototropic curvature of 3-d-old dark-grown Col-0, *pks4-2*, *pks4-2 PKS4* WT-3 and pks4-2 PKS4 F*-1, F*-2, and F*-3 seedlings treated with unidirectional BL. Seedlings were exposed to 0.1 *µ*mol m^−2^ s^−1^ BL for 24 h prior to measurement of growth reorientation. *n* = 40 to 60, different lowercase letters are significantly different (*P* > 0.01, one-way ANOVA with Tukey's HSD test). **D)** Hypocotyl growth orientation of Col-0, *pks4-2*, *pks4-2 PKS4* WT-3 and pks4-2 PKS4 F*-1 and F*-2 seedlings growing in continuous RL (30 *µ*mol m^­2^ s^­1^). Seedlings were kept in darkness for 24 h prior to 4 d of RL exposure, after which growth orientation was measured. 0° represents vertical growth. We consider the absolute value of the angle, whether the seedling bends towards the left or the right side. *n* = 70 to 80, different lowercase letters are significantly different (*P* > 0.01, one-way ANOVA with Tukey's HSD test).

Our analysis of PKS1 truncations fused to GFP and mutating the conserved cysteine residues in motifs C and F revealed that motif C is the primary determinant for PM association (Figs. 4 and 5). However, motifs F and C are related to each other, and we detected less intracellular signal in plants expressing *CDEF-GFP* than *ABC-GFP*; therefore, we decided to determine whether motif F may contribute to tight PM association (Fig. 4B). We, therefore, transformed *pks4* with a construct driven by the *PKS4* promoter and encoding a GFP-tagged PKS4 Cys-to-Ser variant at the invariant Cys residue. In hypocotyl cortex cells of etiolated seedlings, we observed that PKS4 F*-GFP localizes to the cell periphery, as seen earlier for the PKS1 F*-GFP variant (Fig. 5A) and consistent with PM localization (Fig. 8A). To test the functional importance of motif F, we generated another construct driven by the PKS4 promoter and encoding an HA-tagged PKS4 motif F* variant (with the invariant Cys replaced with Ser), which we transformed into *pks4*. We selected transgenic lines (PKS4 F*1-3) with similar protein abundance as in our *PKS4* WT control line (Fig. 8B). We established that all 3 PKS4 F* lines fully complement the phototropic defect of the *pks4* mutant (Fig. 8C). Similarly, these PKS4 F* lines also complemented phytochrome-mediated inhibition of hypocotyl gravitropism in RL and showed a normal hypocotyl gravitropic response in darkness (Fig. 8D and Supplemental Fig. S4C). We conclude that mutating the invariant Cys residue in motif F has no measurable consequences on the light responses analyzed here. Collectively, our data suggest that acylation of the highly conserved Cys residues in motif C is essential for the biological functions of PKS4 and its efficient localization at the PM, while the role of motif F remains to be determined.

### The type of lipid-mediated PKS4 PM association affects its biological activity

**Figure 9.**
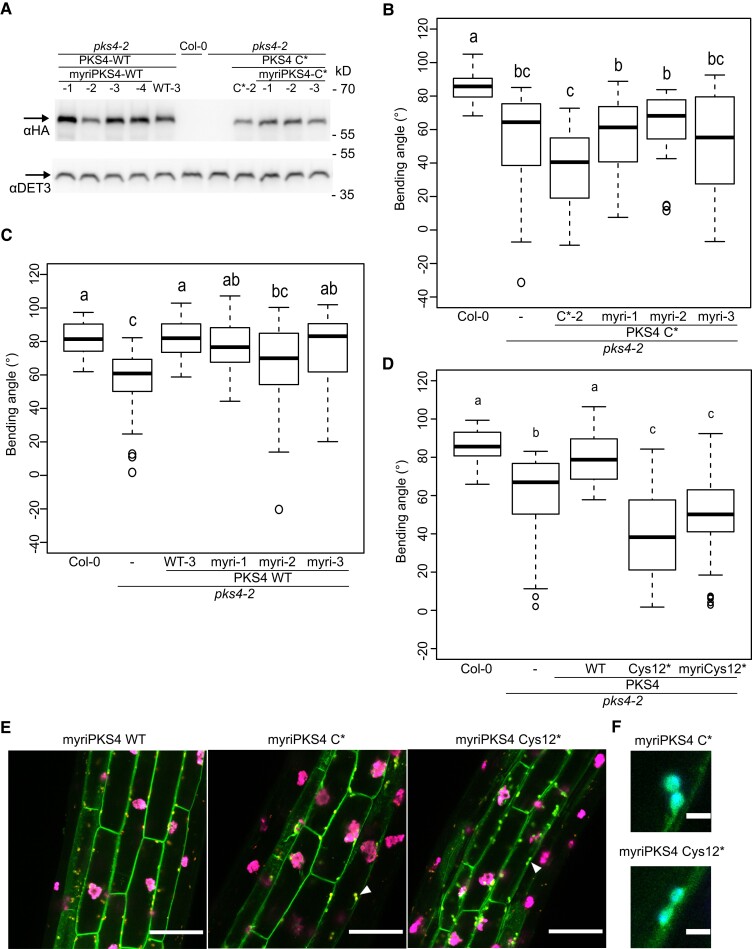
Targeting PKS4 C* to the PM through myristoylation does not rescue PKS4 function. **A)** Immunoblot analysis of HA-tagged PKS4 using an anti-HA antibody on total protein samples extracted from Col-0, *pks4-2*, *pks4-2 PKS4* WT-3, pks4-2 PKS4 C*-2, *pks4-2 myriPKS4* WT-1, -2, -3, -4, myriPKS4 C*-1, -2, and -3 3-d-old dark-grown seedlings. The same membrane was probed with anti-DET3 antibodies as a loading control. **B)** Phototropic curvature of 3-d-old dark-grown Col-0, *pks4-2*, pks4-2 PKS4 C*-2, and pks4-2 myriPKS4 C* (myriC*-1, myriC*-2, and myriC*-3) seedlings treated with unidirectional BL. Seedlings were exposed to 0.1 *µ*mol m^−2^ s^−1^ BL for 24 h prior to measurement of growth reorientation. *n* = 25 to 60, different lowercase letters are significantly different (*P* > 0.01, one-way ANOVA with Tukey's HSD test). **C)** Phototropic curvature of 3-d-old dark-grown Col-0, *pks4-2*, *pks4-2 PKS4* WT-3, and *pks4-2 myriPKS4* WT (*myriWT*-1, *myriWT*-2, and *myriWT*-3) seedlings treated with unidirectional BL. Seedlings were assayed as in **B**. **D)** Phototropic curvature of 3-d-old dark-grown Col-0, *pks4-2*, *pks4-2 PKS4* WT, pks4-2 PKS4 Cys12*, and pks4-2 myriCys12* seedlings treated with unidirectional BL. Primary transformants in the *pks4-*2 background harboring *PKS4* WT and the different variants were assayed. Seedlings were exposed to 0.1 *µ*mol m^−2^ s^−1^ BL for 24 h prior to measurement of growth reorientation. *n* = 30 to 60, different lowercase letters are significantly different (*P* > 0.01, one-way ANOVA with Tukey's HSD test). **E)** Confocal microscopy images of 3-d-old etiolated hypocotyls cortex cells expressing *myriPKS4-GFP*, myriPKS4 C*-GFP, and myriPKS4 Cys12*-GFP (green signal) from the *PKS4* promoter. Note that these lines also show oil bodies (in magenta) resulting from the expression of *OLE1-red fluorescent protein (RFP)* from the *OLE1* promoter, used as a seed coat selection marker. Scale bars, 50 *µ*m. The arrowheads indicate stromules, which are shown in greater magnification in **(F)** Characteristic of stromules, the GFP signal surrounds the blue signal from protochlorophyllide autofluorescence (overlap in cyan). Scale bars, 5 µm.

To determine whether the altered subcellular localization and the biological function of PKS4 C* variants can be rescued by targeting the protein to the PM through another means, we generated transgenic lines in the *pks4* mutant background in which transgene-derived PKS4 (WT and C* variant) was fused to an N-terminal myristoylation (myri) sequence. We selected single insertion lines (*myriPKS4* WT 1-4 and myriPKS4 C* 1-3) with comparable protein levels as in our *PKS4* WT and PKS4 C* lines (Fig. 9A). We discovered that the myriPKS4 C* lines do not complement the phototropism defect of *pks4* (Fig. 9B). However, we observed that myriPKS4 C* lines show a slightly stronger phototropic response than the PKS4 C* line (Fig. 9B). We noticed a similar (but not significant) apparent tendency when comparing numerous independent T1 transformants (Supplemental Fig. S5A). Turning to the intact PKS4 protein fused to the myri sequence, we determined that this addition leads to partial complementation of the phototropic defect seen in *pks4* (Fig. 9C). However, both in the selected T3 lines and when comparing numerous independent T1 transformants, adding the myri sequence to the WT PKS4 protein interfered with its ability to fully complement the phototropic defect of *pks4* (Figs. 9C and Supplemental Fig. S5A). Given that the critical Cys in motif C is Cys-12 (Fig. 7), we also generated a myri version of Cys-12 mutated to Ser, either with an HA tag for complementation or with a GFP tag for subcellular localization assays. The myri-Cys-12-Ser mutant failed to complement the phototropic defect of *pks4* (Fig. 9D). However, as observed for the PKS4 C* variant, the myri-Cys-12-Ser mutant with a myri tag tended (although not significantly) to have a stronger phototropic response than PKS4 C* or Cys-12-Ser alone (Fig. 9, B and D). We then analyzed the subcellular localization of myri-PKS4-GFP, myri-PKS4 C*-GFP, and myriPKS4 Cys12*-GFP in hypocotyl cortex cells of etiolated seedlings and observed all 3 variants to be associated with the cellular periphery (Fig. 9E). In addition, we detected a punctate pattern in those cells, which are most probably stromules, as the GFP signal typically surrounded the autofluorescence produced by protochlorophyllide (Fig. 9F). These structures were easier to visualize using maximal projection images of cortex cells, which showed that they are particularly apparent in the myri C* and Cys-12 variants (Supplemental Fig. S5B). Hence, while the myri tag was able to bring variant proteins with mutation in their C motif to the cellular periphery, it also led to an alteration of PKS4 subcellular localization. Collectively these data indicate that bringing PKS4 C* or Cys12* to the PM through an N-terminal myristoylation sequence is not sufficient to restore its biological activity, while adding this sequence to WT PKS4 moderately interferes with PKS4 function.

Given that bringing PKS4 to the PM through an N-terminal lipid modification appears to perturb PKS4 activity, we tried PM association through the C terminus. Accordingly, we used a farnesylation sequence that was included after the triple HA tag of both PKS4 WT and PKS4 C* and transformed these encoding constructs, driven from the *PKS4* promoter, into the *pks4* mutant. We then compared the phototropic response of numerous independent T1 transformants expressing either *PKS4* WT, PKS4 C*, *PKS4* WT-*farn*, or PKS4 C*-farn constructs. This experiment showed that the farnesylation sequence interferes with the ability of the *PKS4* WT construct to fully complement *pks4* (Supplemental Fig. S5C). The tendency was the opposite in the context of the PKS4 C* mutant, with the farnesylation sequence (C*-farn) displaying a slightly more robust phototropic response than the PKS4 C* construct (Supplemental Fig. S5C). However, the difference in complementation potential of the PKS4 C* and PKS4 C*-*farn* constructs was not significant and neither construct complemented *pks4* (Supplemental Fig. S5C). To determine whether the inclusion of a C-terminal farnesylation sequence restored PM localization of the PKS4 C* variant, we transformed the *pks4* mutant with a construct driven by the PKS4 promoter and encoding a GFP fusion and analyzed stably transformed seedlings. We established that adding a farnesylation sequence after GFP is sufficient to bring PKS4-C*-GFP-farn to the cellular periphery (Supplemental Fig. S5D). In addition, as observed with myri-tagged lines, we observed a punctate pattern both for the WT and C* mutants. Collectively these experiments showed that bringing PKS4 C* to the PM either through an N-terminal myristoylation or a C-terminal farnesylation sequence was not sufficient to restore the ability of the PKS4 C* mutant to complement the phototropic defect of *pks4*. However, these results are difficult to interpret given that these modifications also altered the complementation potential of WT PKS4 and led to some alteration of PKS4 subcellular localization with enhanced localization to stromules.

## Discussion

We identified PKS sequences in seed plants but not in the genomes of mosses, ferns, liverwort, or algae, indicating that these proteins appeared relatively late in land plant evolution (Fig. 1 and Supplemental Fig. S2). This evolutionary history is similar to that of BG proteins, which share sequence motifs with PKS proteins (Fig. 3) (Mishra et al. 2017). By contrast, members of the NRL family are present in all land plants, while phototropin-type photoreceptors are present in land plants, charophytes, and chlorophytes (Christie et al. 2018). PKS proteins also appear much later than phytochromes, which are found in charophytes and land plants (not considering phytochromes in cyanobacteria, bacteria, or fungi) (Li and Mathews 2016). Based on this evolutionary history, it is tempting to propose that PKS proteins are required for a seed-plant-specific function and/or were required following changes in the light environment triggered by the expansion of seed plants (Li and Mathews 2016). While we detected a single PKS sequence in some gymnosperms, basal angiosperms have 2 members of the family that we call PKS1 and PKS4 based on the Arabidopsis nomenclature (Fig. 1 and Supplemental Fig. S2).

PKS proteins share 6 or 7 sequence motifs that are present in the same order and separated by regions of various lengths and without obvious similarity (Fig. 2). We previously showed that PKS4 is phosphorylated in a light and phot1-dependent manner on Ser-299 (Schumacher et al. 2018). This residue is located between motifs D and E. The protein motifs identified here are unique to PKS proteins, except for motifs D and A, which are also present in BG proteins (Fig. 3) (Mishra et al. 2017). The molecular function of BG proteins is largely unknown; however, similar to PKS proteins, they are thought to regulate auxin transport and/or signaling (de Carbonnel et al. 2010; Kami et al. 2014; Liu et al. 2015). Additional experiments are required to determine whether and how these proteins perform such functions and whether the common sequence motifs are involved in controlling the distribution of auxin.

The PM is the major site of phototropin action, where these photoreceptors are part of a protein complex comprising PKS proteins (Lariguet et al. 2006; de Carbonnel et al. 2010; Demarsy et al. 2012; Preuten et al. 2015). We, therefore, tested whether any of the conserved PKS motifs are required for PM association. We established that motif C is a major determinant for the subcellular localization of PKS1 and PKS4 (Figs. 4 and 5). Given that PKS1 and PKS4 are representatives of both PKS clades present in angiosperms (Fig. 1), this result suggests that the function of motif C is broadly shared among PKS proteins. Motif C contains highly conserved Cys residues, particularly Cys-12 (12th amino acid of the motif) and to a lesser extent Cys-10 (Fig. 2). Mutating all 3 conserved Cys residues in PKS1 motif C prevented protein acylation in vivo and localization of the protein to the PM (Fig. 5). PKS4 has 2 Cys residues in motif C (Cys-10 and Cys-12). Mutating both residues increased intracellular signaling and decreased PM association of the GFP-tagged variant; based on variants mutated in either Cys-10 or Cys-12 showed that the most highly conserved Cys-12 residue is critical (Fig. 7). This result suggests that S-acylation of Cys-12 is essential for PKS association with the PM. Interestingly, motif F, which is related to motif C, does not play a prominent role in the control of PKS protein localization (Figs. 2B and 8). A recent large-scale analysis of protein acylation in Arabidopsis identified PKS1, PKS2, and PKS3 as S-acylated proteins (Kumar et al. 2022). Moreover, acylation of PKS proteins was mapped to Cys residues in motif C and in motif F (Kumar et al. 2022), independently confirming our data on the S-acylation of motif C in PKS1. PKS4 was not identified in this earlier study (Kumar et al. 2022), possibly because the authors used light-grown seedlings and PKS4 protein levels decline rapidly in etiolated seedlings transferred into the light (Demarsy et al. 2012). While S-acylation is very difficult to predict based on primary amino-acid sequence, lysine residues are often found in the vicinity of S-acylated cysteines (Zaballa and van der Goot 2018). For some proteins, S-acylation was shown to prevent ubiquitination of the nearby lysine and subsequent protein degradation (Zaballa and van der Goot 2018). We note that in motif F there is an invariant lysine residue that is much less conserved in motif C (Fig. 1B). One possibility is that motif F regulates the stability of PKS proteins. In summary, our data are consistent with S-acylation of highly conserved Cys residues of motif C playing a key role in PM localization of PKS proteins, while the role of motif F requires further investigation.

By testing the ability of PKS4 variants to complement phototropism and hypocotyl gravitropism of *pks4* mutants, we established that variants impaired in PM localization also fail to complement *pks4* (Figs. 6 and 7). Biochemical fractionation indicated that PKS4 C* is not cytosolic but membrane-associated (in the microsome faction) (Fig. 7A). The nature of these intracellular membranes requires further investigation. Moreover, this PKS4 C* variant still interacted with NPH3 (Fig. 7B). However, we found reduced levels of the light-induced PKS4 isoform in the mislocalized PKS4 C* variant (Fig. 6C). This observation is consistent with PKS4 being a phototropin signaling element acting at the PM and a substrate of phot1 kinase activity (Demarsy et al. 2012). Collectively, these data suggest that PKS4 is a component of phototropin and phytochrome signaling that acts at the PM (Schepens et al. 2008; Kami et al. 2014). While such a localization is expected for a protein acting downstream of phototropins, this result contrasts with most well-characterized phytochrome signaling events, which occur in the nucleus (Legris et al. 2019; Cheng et al. 2021).

To further test the link between PKS4 subcellular localization and its ability to regulate hypocotyl growth orientation, we attempted to tether PKS4 C* variants to the PM by adding either an N-terminal myristoylation sequence or a C-terminal farnesylation sequence. Both approaches partially restored the subcellular localization of PKS4 C* and Cys-12* (Fig. 9 and Supplemental Fig. S5). Moreover, we noticed that the addition of a myristoylation sequence triggered the association of these proteins with stromules (Fig. 9). These structures were particularly prominent when the tag was added to C* variants (Fig. 9 and Supplemental Fig. S5). In addition, these PKS4 variants were not able to complement the phototropic defect of *pks4* (Fig. 9 and Supplemental Fig. S5). Interestingly, phototropism in seedlings expressing the PKS4 C* variant was often less efficient than in *pks4*, suggesting that accumulation of the mislocalized PKS4 C* interferes with phototropism (Figs. 6B and 9B and Supplemental Fig. S5). Consistent with this idea, partial restoration of PKS4 C* localization to the PM (through myristoylation or farnesylation of PKS4 C*) reduced the severity of the phototropic defect (Fig. 9 and Supplemental Fig. S5). Nevertheless, these variants were unable to complement the phototropic defect of *pks4* (Fig. 9 and Supplemental Fig. S5). Moreover, including a myristoylation or farnesylation sequence to PKS4 slightly interferes with the ability of wild-type PKS4 to promote phototropism (Fig. 9 and Supplemental Fig. S5). A possible explanation for these observations is that how PKS4 attaches to the PM influences its ability to work in phototropin signaling. Indeed, the wild-type protein is attached to the PM through the middle of the protein, which is a distinguishing feature of S-acylation contrasting with N-terminal or C-terminal lipid-mediated PM association occurring through myristoylation or farnesylation (Turnbull and Hemsley 2017; Hemsley 2020). PKS4 with myristoylation or farnesylation tags is expected to associate with the PM through the C motif and the N or C terminus, but PKS4 C* with additional lipid modification sequences is expected to associate with the PM only through the N or the C terminus. This difference in protein attachment to the PM may explain the observed phenotypes, for example, because the N and C termini must be free to efficiently engage in protein–protein interactions. Alternatively, while S-acylation is reversible and allows regulated association with the PM, myristoylation or farnesylation are irreversible modifications (Hemsley 2020). In the case of NPH3, its cycles of PM association and dissociation are functionally important (Sullivan et al. 2021). We do not have evidence for regulated PKS subcellular localization. Moreover, adding either an N-terminal myristoylation or a C-terminal farnesylation sequence to PKS4 only interfered slightly with the ability of the encoding protein to complement *pks4* (Fig. 9 and Supplemental Fig. S5). Therefore, our current data do not provide evidence for regulated, S-acylation-mediated PKS localization playing a key functional role. Instead, we propose that proper positioning of PKS to the PM is functionally important, but we cannot rule out that Cys-to-Ser mutations in the conserved C motif altered PKS4 activity through a yet-to-be-discovered mechanism.

## Materials and methods

### PKS phylogeny and motif discovery

The PKS sequences used to construct the tree were obtained from multiple sources. Most sequences were obtained from OMA Hierarchical Orthologous Groups (HOGs) from the January 2020 release of the OMA browser (Altenhoff et al. 2021). The first 3 HOGs were found by searching the browser for protein sequences from *A. thaliana PKS1*, *PKS2*, *PKS3*, and *PKS4* genes (At2g02950, At1g14280, At1g18810, and At5g04190). *PKS1* and *PKS2* were inferred by OMA to be in the same gene family (rooted at the Magnoliopsida level), and *PKS3* and *PKS4* were in 2 other families, rooted at the Pentapetalae and Mesangiospermae levels, respectively. A final, smaller gene family was found in OMA by searching for the *A. trichopoda PKS4* gene, which was rooted at the Magnoliopsida level. Seventeen additional *PKS* sequences not present in the OMA database, selected to increase phylogenetic diversity, were added to cover the following: the basal angiosperms *A. trichopoda* and *N. colorata*; the monocots purple false brome (*Brachypodium distachyon*) and foxtail millet (*Setaria italica*); the magnoliid avocado (*P. americana*); the asterids potato (*Solanum tuberosum*) *and* tea plant (*Camellia sinensis*); the rosid wild strawberry (*Fragaria vesca*); and the Caryophyllales sugar beet (*Beta vulgaris*). The aforementioned sequences were obtained by reciprocal BLAST searches using Arabidopsis PKS1 and PKS4 protein sequences as query (Altschul et al. 1990). Two gymnosperm *PKS-LIKE* genes were found from PLAZA Gymnosperms 1.0 (Proost et al. 2015). Thus, a total of 172 PKS homologs (protein sequences) were used for the remainder of the analysis (Supplemental Data Set 1).

The 172 sequences were aligned using MAFFT v7.313 (Katoh and Standley 2013), with the E-INS-i algorithm option. This algorithm was chosen because it uses a “generalized affine gap cost” in the pairwise alignment stage, which is better to use for sequences with long unalignable regions, such as in PKS proteins. The alignment was then filtered to remove unreliable columns in the alignment matrix: those with gaps in more than 20% of the sequences (gap threshold 0.8), essentially those columns not containing highly conserved motifs, resulted in 250 columns in the final alignment.

The alignment was used to make a gene tree of all PKS sequences using IQ-TREE web server version 1.6.12 (Trifinopoulos et al. 2016) with the ModelFinder (Kalyaanamoorthy et al. 2017), tree reconstruction (Nguyen et al. 2015), and ultrafast bootstrap (1,000 replicates) (Hoang et al. 2018) options. Ultrafast bootstrap was implemented because it has been shown to be orders of magnitude faster to compute while maintaining accurate equivalents to standard bootstrap methods (Minh et al. 2013; Hoang et al. 2018). The resulting maximum-likelihood tree was visualized with phylo.io (Robinson et al. 2016), and manually rooted using gymnosperm sequences as an outgroup. The unfiltered alignment (i.e. before trimming) and phylogenetic tree are provided as Supplemental Files 1 and 2.

To identify motifs in the highly gapped alignment, GLAM2 was utilized (Frith et al. 2008). Since GLAM2 can only find 1 motif per alignment, we trimmed the sequences based on manual inspection of the alignment as well as gblocks conserved locations (Talavera and Castresana 2007) and included the flanking 20 amino acids on each side. The parameters were set to “default”, except the initial columns to be aligned were set to 10, the maximum columns to be aligned were set to 20, and to shuffle the sequences.

To compare motifs C and F and report a statistical measure of motif similarity, we used the Tomtom webserver (https://meme-suite.org/meme/tools/tomtom) (Gupta et al. 2007). The Sandelin-Wasserman similarity was chosen for the column comparison function. To run the comparison, we produced a database of GLAM2 output for all PKS motifs and searched motifs C and F separately against the database. Only motif comparisons with a *P*-value of <0.05 were considered statistically significant.

To search for motifs common between BIG GRAIN and PKS, we used MEME, an algorithm and webserver to find ungapped motifs in protein sequences (https://meme-suite.org/meme/tools/meme) (Bailey et al. 2015) The BIG GRAIN protein sequences from Arabidopsis and other plant species were obtained from OMA HOGs (Altenhoff et al. 2021) using Arabidopsis identifiers from Mishra et al. (2017). We combined all BIG GRAIN and PKS protein sequences to search for motifs using MEME with default parameters except for 0 or 1 motif occurrence per sequence and a maximum of 15 motifs.

### Plant materials

All plants utilized in this study are in the Arabidopsis (*A. thaliana*) Columbia-0 accession. The *pks4-2* allele (GABI_312E01) was utilized in this study (Schepens et al. 2008). *35S:PKS1-GFP* (pCF202), *35S:PKS4-GFP* (pIS03), and *PKS4pro:PKS4-3XHA* (pPS09) in *pks4-2* were previously described (Lariguet et al. 2006; Demarsy et al. 2012; Schumacher et al. 2018). To obtain constructs driven by the 35S promoter and encoding PKS1 truncations fused to GFP, *PKS1* amplicons were cloned using KpnI-BamHI restriction sites into a binary vector designed to generate C-terminal GFP fusions (pCF203). The *PKS1* cDNA from plasmid pCF173 was amplified with the following primer combinations (CF129/CF470) corresponding to amino acids (aa) 1–160 of PKS1 (AB); (CF129/CF471) corresponding to aa 1-273 (ABC); (CF472/CF473) corresponding to aa 274-439 (DEF); (CF507/CF471) corresponding to aa 161-273 (C), and (CF507/CF473) corresponding to aa 161-439 (CDEF). The PCR products were digested with Kpn1 and BamH1 and ligated into digested pCF203 to obtain pCF524 (AB), pCF525 (ABC), pCF526 (DEF), pCF534 (C), and pCF535 (CDEF). Mutations of the 3 conserved Cys residues in motif C were obtained by site-directed mutagenesis using pCF173 as a template to generate pCF546 (ABC*), pCF547 (ABC*DEF), and pCF550 (C*DEF). Mutations of the conserved Cys residues in motif F (Cys-378) were also obtained by site-directed mutagenesis using pCF173 as a template to obtain pCF393. For PKS4 C* lines, a fragment encoding PKS4 ABC (aa 1 to 186 of PKS4 with Cys-143 and Cys-145 mutated to Ser) was ordered at Eurofins and after digestion with XmaI/Bpu10I was ligated into a *35S:PKS4-GFP* construct to replace the wild type with the mutant sequence to obtain pCF561. For the generation of the PKS4pro:PKS4 C*-3xHA lines (pAL10), a 517-bp DNA fragment containing *PKS4* encoding the C143S and C145S variants was digested from pCF561 with the restriction enzymes EcoRV and NruI and replaced in the pPS9 construct (Schumacher et al. 2018) previously digested with the same restriction enzymes to replace the wild type with the mutant sequence. For the generation of the PKS4pro:PKS4 Cys10*-3xHA and PKS4pro:PKS4 Cys12*-3xHA lines (pAL79 and pAL80, respectively), a synthetic 517-bp DNA fragment of the *PKS4* coding sequence harboring the C143S or C145S mutation and flanked by the EcoRV and NruI sites was ordered from Eurofins and replaced in the pPS9 construct previously digested with the same restriction enzymes to replace the wild type with the mutant sequence. Similarly, for the generation of the PKS4pro:PKS4 F*-3xHA lines (pAL40), a synthetic 682-bp DNA fragment of the *PKS4* coding sequence carrying the C358S mutation and flanked by the EcoRV and BamHI sites was ordered from Eurofins and replaced in the pPS9 construct previously digested with the same restriction enzymes to replace the wild type with the mutant sequence. For the generation of the *PKS4pro:myriPKS4-3xHA* (pAL63) and PKS4pro:myriPKS4 C*-3xHA (pAL64) and PKS4pro:myriPKS4 Cys12*-3xHA (pAL86) lines, a synthetic 171-bp DNA fragment of the *PKS4* coding sequence containing the last part of the *PKS4* promoter and the myristoylation signal sequence ATGGGAATTTGTATGTCTAGA followed by the beginning of the *PKS4* coding sequence was ordered and digested with the restriction enzymes XhoI and NruI and replaced in the pPS9, pAL10, and pAL80 constructs previously digested with the same restriction enzymes. For the generation of the *PKS4pro:PKS4-3xHAfarn* (pAL70) and PKS4pro:PKS4 C*-3xHAfarn (pAL71) lines, a synthetic 225-bp DNA fragment including the last part of the *PKS4* coding sequence, the *3xHA* tag sequence followed by the farnesylation sequence TCT AAG GAT GGA AAG AAG AAG AAG AAG AAG TCT AAG ACT AAG TGT GTT ATT ATG, and a very short fragment of the backbone vector flanked by the unique restriction enzymes sites BamHI and PstI was replaced in the pPS9 and pAL10 previously digested with the same restriction enzymes. For the generation of *PKS4pro:PKS4-GFP* (pAL43) lines, a DNA fragment containing the *PKS4 pro:PKS4-GFP:term* in pED10 (Demarsy et al. 2012) was digested with the restriction enzyme HindIII and cloned into pFR100 (pFP100-based vector [Bensmihen et al. 2004] carrying the *OLE1pro:OLE1-FastRed* for selection of transgenic plants) previously digested with the same enzyme. For the generation of the PKS4pro:PKS4 C*-GFP (pAL45) and PKS4pro:PKS4 F*-GFP (pAL65), PKS4pro:PKS4 Cys10*-GFP (pAL81), and PKS4pro:PKS4 Cys12*-GFP (pAL82) lines, a DNA fragment encoding PKS4 with C143S and C145S, or C358S, or C143S, or C145S mutations, respectively, was digested from the pAL10, pAL40, pAL79, and pAL80 vectors with the restriction enzymes NruI and ZraI and replaced in pAL43 previously digested with the same enzymes. For the generation of the *PKS4pro:myriPKS4-GFP* (pAL61), PKS4pro:myriPKS4 C*-GFP (pAL62), and PKS4pro:myriPKS4 Cys12*-GFP (pAL87) lines, the same synthetic 171-bp DNA fragment used for the generation of pAL63 and pAL64 was digested with the restriction enzymes XhoI and NruI and replaced in the pAL43, pAL45, and pAL82 previously digested with the same restriction enzymes. For the generation of the *PKS4pro:PKS4-GFPfarn* (pAL67) and PKS4pro:PKS4 C*-GFPfarn (pAL68) lines, the same synthetic 1,557-bp DNA fragment used for the generation of pAL70 and pAL71 was replaced in pAL43 and pAL45 previously digested with the same restriction enzymes. All constructs were verified by Sanger sequencing. Primers are provided in Supplemental Table S1. Transgenic lines were obtained using Agrobacterium (*Agrobacterium tumefaciens*)-mediated transformation with Agrobacterium GV3101 (Bent 2006). Several single insertion lines were characterized. The use of fluorescent seeds as selection markers also allowed us to perform experiments with large numbers of independent T1 transformants.

### Growth conditions

For seed production, plants were grown on soil at 22 °C with 16 h of white light (WL) per day. For physiological experiments, seeds were surface-sterilized in 70% (v/v) ethanol and 0.05% (v/v) Triton-X for 5 min and in 100% ethanol for 5 min. Seeds were then sown on Petri plates containing half-strength MS medium, pH 5.7, 0.8% (w/v) agar. Plates were stored in the dark for 3 d at 4 °C for stratification. For experiments with dark-grown seedlings, germination was induced by 4 to 6 h of WL (80 *μ*mol m^−2^ s^−1^) at 22 °C, and plates were placed back in the dark at 19 °C or 22 °C for 3 d before light exposure. For inhibition of gravitropism experiments, germination was induced by 1 h of RL (50 *μ*mol m^−2^ s^−1^) at 22 °C and plates were placed back in the dark at 22 °C for 1 d before light exposure.

### Light treatments

For growth in the dark, seedlings were grown on vertically oriented plates for 3 d in darkness at 19 °C or 22 °C prior to light treatment. For phototropism assays, seedlings were irradiated with constant unilateral 0.1 *µ*mol m^−2^ s^−1^ BL at 22 °C for up to 24 h; for protein extraction, seedlings were irradiated with unilateral 1 *µ*mol m^−2^ s^−1^ BL at 22 °C during 0, 1, 3, 10, and 20 min. For inhibition of gravitropism, seedlings were grown on vertically oriented plates for 1 d in darkness at 22 °C prior to light treatment. Seedlings were irradiated with constant 30 *µ*mol m^−2^ s^−1^ RL at 22 °C for 3 d.

### Hypocotyl measurements and analysis

Plates were pictured using an infra-red CCD camera system at different time points. The curvature angles were calculated by subtracting the average angle of orientation of the upper region (85% to 95% of total length) of each hypocotyl with respect to the vertical after light treatment, as determined by a customized MATLAB script developed in the Fankhauser Lab. One-way ANOVA (aov) and Compute Tukey's Honest Significance Differences (HSD.test) [agricolae package] were performed in R. We considered *P*-values <0.01 significant.

### Fluorescence microscopy

Confocal microscopy images were taken with a confocal microscope model LSM 510 (Zeiss), LSM 880 (Zeiss), or Stellaris 5 (Leica). Model LSM 510 was used for all PKS1 imaging and for *35S:PKS4-GFP* and 35S:PKS4_C*- GFP. Excitation was accomplished using an Argon laser at 488 nm and detection used a bandpass emission between 505 and 530 nm. For some images, the PM was stained with the dye FM4-64 (Cat. T13320, Invitrogen) at a concentration of 50 *μ*M, by soaking the seedlings for 1 min and then washing 3 times in liquid half-strength MS medium. In that case, the signal was detected with a long pass emission from 650 nm. Model LSM 880 was used for imaging PKS4pro:PKS4 F*-GFP and PKS4pro:PKS4 C*-GFPfarn. Samples were excited with an argon laser (488 nm) and detection was done between 495 and 518 nm for GFP and between 607 and 691 nm for chlorophyll and red fluorescent protein (RFP). Leica Stellaris 5 was used to image *PKS4pro:PKS4-GFP*, PKS4pro:PKS4 C*-GFP, PKS4pro:PKS4 Cys10*-GFP, PKS4pro:PKS4 Cys12*-GFP, *PKS4pro:myriPKS4-GFP*, PKS4pro:myriPKS4 C*-GFP, PKS4pro:myriPKS4 Cys12*-GFP. The objective used was HC PL APO 63×/1.40 OIL. Samples were excited with a 488-nm laser and fluorescence was detected between 496 and 520 nm for GFP, 580 to 585 nm for RFP, and 670 to 690 nm for protochlorophyllide. For each plant, between 30 and 40 images were taken at different *z* positions, every 1 *µ*m starting in the epidermis and finishing in the second cortex layer. Images were taken below the apical hook, where *PKS4* expression is the highest. Images were edited using ImageJ. In Figs. 7, C and D, 9E, and Supplemental Fig. S4D, pictures are maximal intensity projections of 3 slices. In Supplemental Fig. S5B, pictures are maximal intensity projections of 12 to 18 slices.

### Biotin switch assay

Palmitoylation of the PKS1 fragments was assayed as described in Hemsley et al. (2008). The principle of this method is to first block all free Cys residues in the protein extract. Second, the thioester bonds are selectively cleaved using hydroxylamide at a neutral pH. Third, the newly freed thiol groups are biotinylated and biotinylated proteins are purified by neutravidin-based capture. We included the following small modifications to the method of Hemsley et al. (2008). Three-day-old etiolated seedlings were ground in a cold mortar at 4 °C and resuspended in 2 × volume of lysis buffer per fresh weight. After the first centrifugation, 1 mL of the supernatant was combined with 1 mL of lysis buffer and incubation was done for 3 h at 4 °C on a roller table. All centrifugation steps were performed at 4 °C. The loading control was not precipitated. We used 60 *µ*L of high capacity neutravidin-agarose beads (Thermo Fisher) instead of 15 *µ*L.

### Cell fractionation

One thousand seeds per replicate (100 *µ*L in an Eppendorf tube) were sown onto plates containing half-strength MS medium solidified with 08% (w/v) agar. After stratification for 3 to 5 d, germination was induced with 6 h of WL (80 *μ*mol m^−2^ s^−1^). Individual plates were incubated at 22 °C for 3 d in darkness. Seedlings were collected, weighted, and ground with a mortar and pestle on ice. Two volumes of extraction buffer (50 mM HEPES pH 7.9, 300 mM sucrose, 150 mM NaCl, 10 mM potassium acetate, 5 mM EDTA, protease inhibitor cocktail P9599-5ML [Sigma]) were added. Samples were centrifuged for 5 min at 1600 rpm (200 g) in a benchtop centrifuge at 4 °C. The supernatant corresponds to the total fraction, S1. The supernatant was centrifuged again for 75 min at 100,000 × *g* at 4 °C. The supernatant corresponds to the soluble fraction, S2. The pellet was resuspended in 1 volume of extraction buffer with 0.5% (v/v) Triton X-100 and centrifuged again for 5 min at 14,000 rpm (15,000 × g) in a benchtop centrifuge at 4 °C. The supernatant corresponds to fraction S3.

### Co-IP

Co-IP was done using a Miltenyi µMACS HA isolation kit (130-091-122), μ Columns (130-042-701), and a μMACS Separator (130-042-602) following the manufacturer's guidelines with a few modifications. The microsomal fraction S3 was used for the Co-IP. Briefly, 340 *µ*L of protein extract was incubated with 50 *µ*L of magnetic beads for 90 min at 4 °C. The first 4 washes were done with extraction buffer (as defined above) + Triton X-100. Elution was done with 2× Laemmli buffer.

### Immunoblotting

For the biotin switch assay, proteins extracted in 2× Laemmli buffer were separated on 12% SDS/PAGE gels and transferred onto nitrocellulose membrane in CAPS buffer. Cell fractionation samples and Co-IP samples were mixed with 1 volume of 2× Laemmli buffer. For the cell fractionation assay, twice as much was loaded of S1 and S2 than S3. For all other immunoblots, total proteins were extracted in Laemmli buffer (80 *µ*L 2× Laemmli buffer for 20 mg fresh weight) and 10 *µ*L was loaded per lane. Proteins were separated on 4% to 15% precast polyacrylamide gels except for data shown in Fig. 6C and Supplemental Fig. S4A, which were separated on larger 10% homemade polyacrylamide gels to allow for greater separation. Proteins were transferred onto nitrocellulose membranes using a Trans-Blot Turbo RTA Transfer Kit. Antibodies were diluted in 1× phosphate buffered saline containing 0.1% (v/v) Tween-20 and 5% (w/v) nonfat dry milk. Anti-HA-HRP monoclonal antibody 3F10 was used at 1/4,000 (12013819001, Roche), anti-GFP monoclonal antibody JL-8 (632381, Clontech) was used at 1/3,000, anti-DET3 antibody was used at 1/20,000 dilutions (Schumacher et al. 1999), anti-CFBPase (AS04043, Agrisera) was used at 1/5,000, and anti-NPH3 (Motchoulski and Liscum 1999) was used at 1/3,000. A chemiluminescence signal was generated using Immobilon Western HRP Substrate (Millipore). The signal was detected with a Fujifilm ImageQuant LAS 4000 mini CCD camera system and quantifications were performed with ImageQuant TL software (GE Healthcare).

### Statistical analysis

Statistical methods are specified in each figure legend. Statistical data are provided in Supplemental Table S2.

### Accession numbers

The Arabidopsis Genome Initiative numbers for the genes mentioned in this article are as follows: *PKS1* (At2g02950), *PKS2* (At1g14280), *PKS3* (At1g18810), *PKS4* (At5g04190), *NPH3* (At5g64330), *BG1* (At5g12050), *BG2* (At3g42800), *BG3* (At1g54200), *BG4* (At3g13980), *BGL1* (At1g69160), *BGL2* (At1g13670).

## Supplementary Material

koad096_Supplementary_Data

## Supplemental data

The following materials are available in the online version of this article.

**Supplemental Figure S1.** PKS proteins are intrinsically disordered.

**Supplemental Figure S2.** Extended PKS protein phylogeny.

**Supplemental Figure S3.** Alignments of Motif C of PKS1 and PKS4 proteins.

**Supplemental Figure S4.** Further characterization of motif C and motif F mutants (C* and F*).

**Supplemental Figure S5.** Targeting PKS4 C* to the PM through farnesylation does not rescue PKS4 function.

**Supplemental Table S1.** Primers used in this study.

**Supplemental Table S2.** Summary of statistical analyses.

**Supplemental Data Set 1.** PKS sequences used in the phylogenetic analysis.

**Supplemental File 1.** Multiple sequence alignment for the tree shown in Fig. 1A and Supplemental Fig. S2.

**Supplemental File 2.** Newick format of the tree shown in Fig. 1A and Supplemental Fig. S2.
