# Supplementary material for: Protein S-acylation controls the subcellular localization and biological activity of PHYTOCHROME KINASE SUBSTRATE
Source: Plant Cell. 2023 Mar 28;35(7):2635–53. doi: 10.1093/plcell/koad096 (PMC10291038; doi:10.1093/plcell/koad096)
Supplement: koad096_Supplementary_Data [file koad096_supplementary_data.zip › tpc.22.00662 Supplemental figures and tables.pdf]

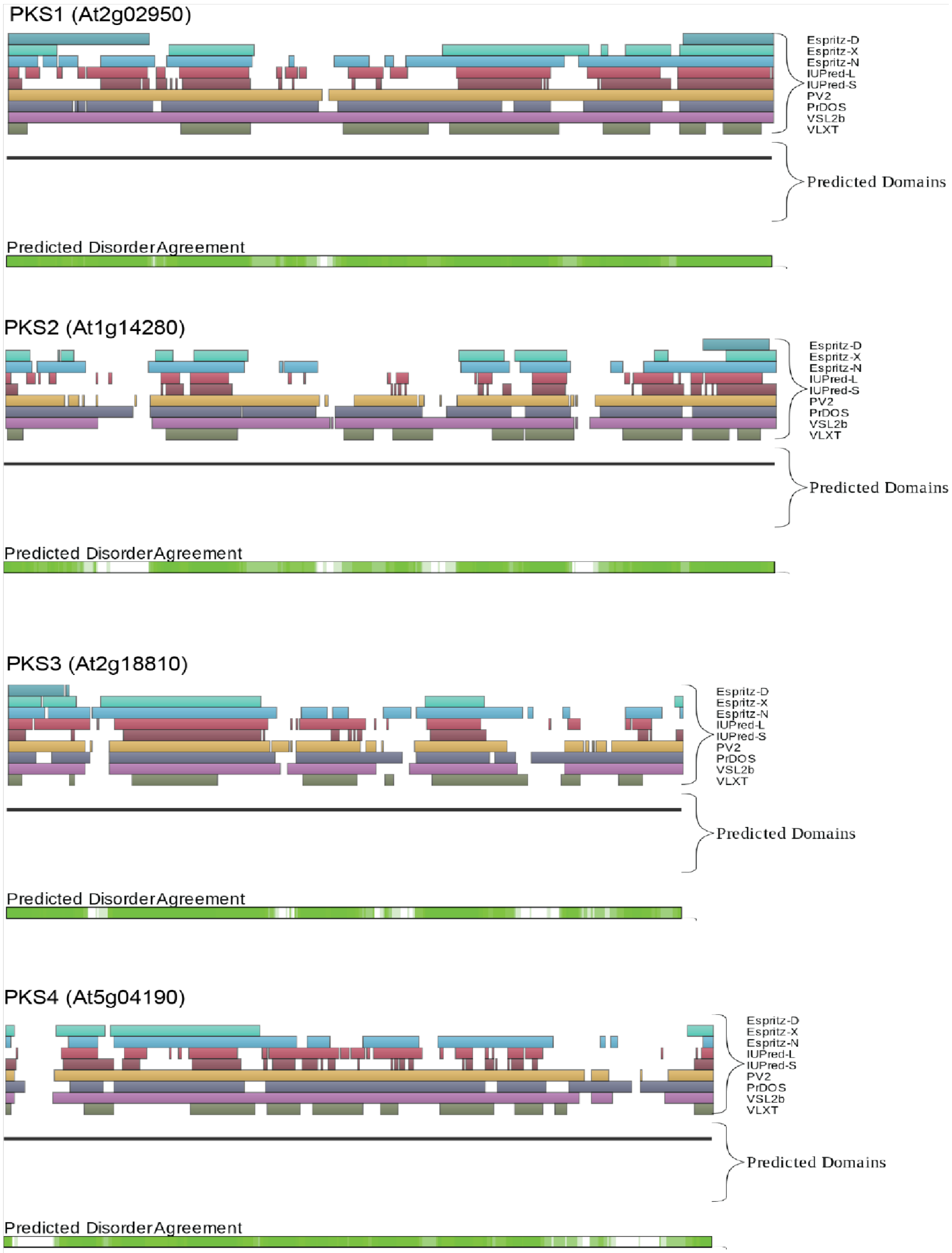

Supplemental Figure S1. PKS proteins are intrinsically disordered. Supports Figure 1.

Disorder propensity of Arabidopsis PKS1-PKS4 as predicted using D^2P^2. This predictor compiles the prediction using several algorithms indicated as boxes of different colors. The last box shows predicted disorder agreement in green. Note that this predictor also fails to identify any known protein domain in PKS1-PKS4.

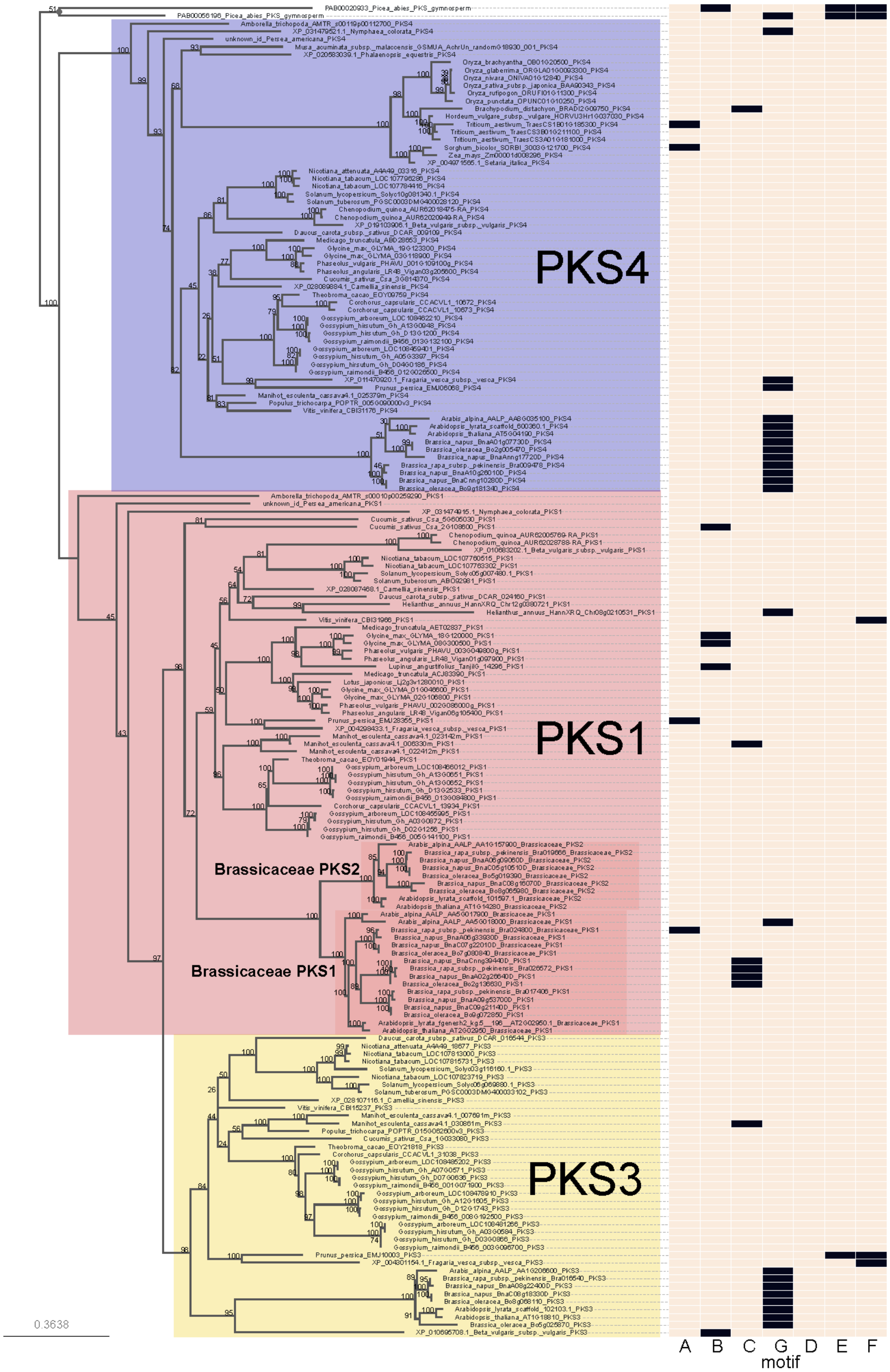
PKS4
PKS1
Brassicaceae PKS2
Brassicaceae PKS1
PKS3
0.3638
A B C G D E F
motif

17

Supplemental Figure S2. Extended PKS protein phylogeny. Supports Figure 1.

Phylogeny of PKS proteins. The tree was obtained using IQ-TREE and the JTT+I+G4 substitution model. The orange, green, and blue boxes highlighting the sequences indicate subfamilies of PKSs 1/2, 3, and 4, respectively. Ultrafast bootstrap values are indicated at the internal nodes. The table to the right of the tree corresponds to the phylogeny: each row in the table corresponds to the sequence to its left, and each column is a motif. A black box in the table indicates absence of a motif in a given sequence. The alignment and tree files are provided as Supplemental Files 1 and 2.

A

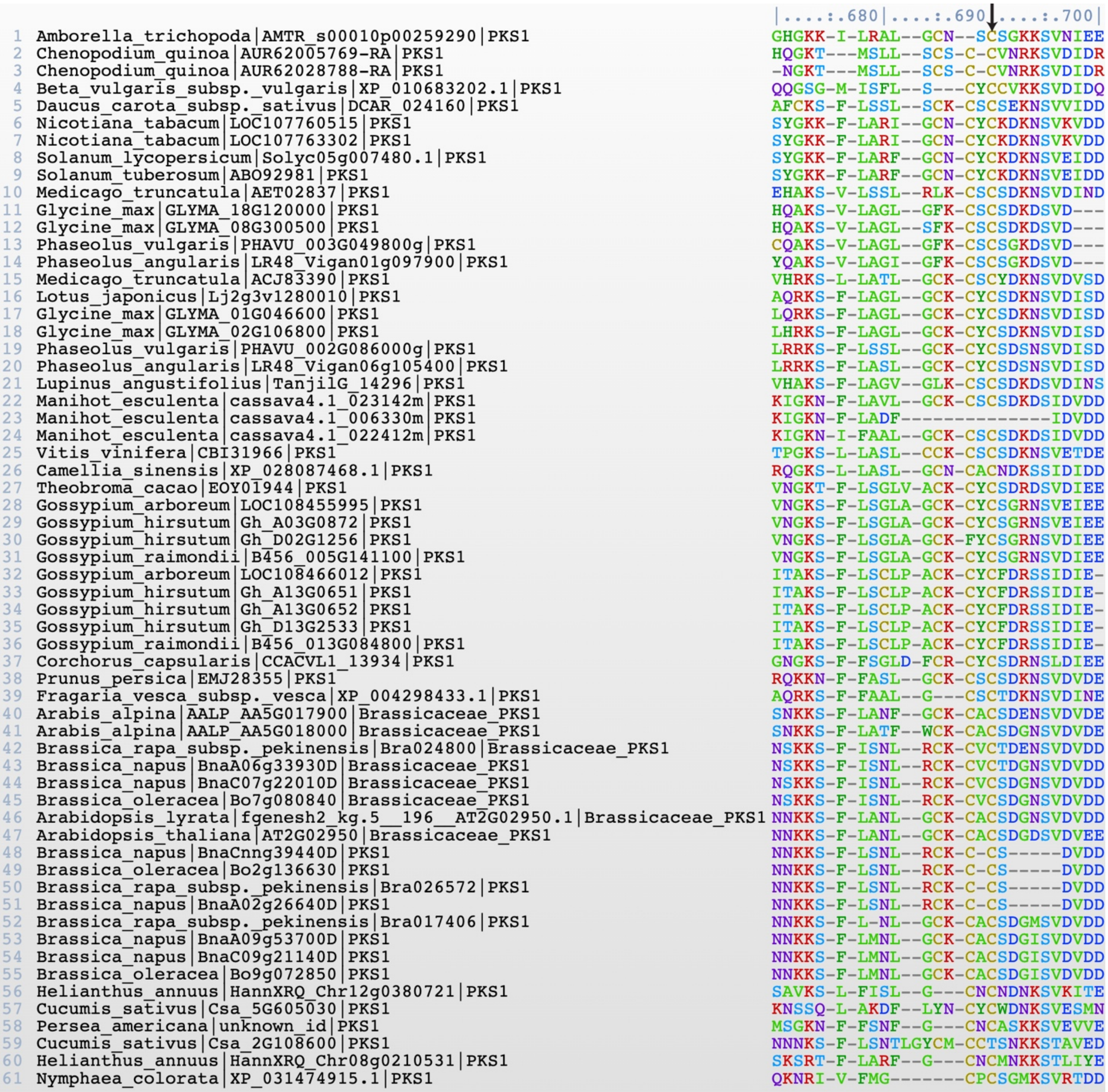
|....:.680|....:.690|....:.700|
1 Amborella_trichopoda|AMTR_s00010p00259290|PKS1 GHGKK-I-LRAL--GCN--SCSGKKSVNIEE
2 Chenopodium_quinoa|AUR62005769-RA|PKS1 HQGKT---MSLL--SCS-C-CVNRKSVDIDR
3 Chenopodium_quinoa|AUR62028788-RA|PKS1 -NGKT---MSLL--SCS-C-CVNRKSVDIDR
4 Beta_vulgaris_subsp._vulgaris|XP_010683202.1|PKS1 QQGSG-M-ISFL--S---CYCCVKKSVDIDQ
5 Daucus_carota_subsp._sativus|DCAR_024160|PKS1 AFCKS-F-LSSL--SCK-CSCSEKNSVVIDD
6 Nicotiana_tabacum|LOC107760515|PKS1 SYGKK-F-LARI--GCN-CYCKDKNSVKVDD
7 Nicotiana_tabacum|LOC107763302|PKS1 SYGKK-F-LARI--GCN-CYCKDKNSVKVDD
8 Solanum_lycopersicum|Solyc05g007480.1|PKS1 SYGKK-F-LARF--GCN-CYCKDKNSVEIDD
9 Solanum_tuberosum|ABO92981|PKS1 SYGKK-F-LARF--GCN-CYCKDKNSVEIDD
10 Medicago_truncatula|AET02837|PKS1 EHAKS-V-LSSL--RLK-CSCSDKNSVDIND
11 Glycine_max|GLYMA_18G120000|PKS1 HQAKS-V-LAGL--GFK-CSCSDKDSVD---
12 Glycine_max|GLYMA_08G300500|PKS1 HQAKS-V-LAGL--SFK-CSCSDKDSVD---
13 Phaseolus_vulgaris|PHAVU_003G049800g|PKS1 CQAKS-V-LAGL--GFK-CSCSGKDSVD---
14 Phaseolus_angularis|LR48_Vigan01g097900|PKS1 YQAKS-V-LAGI--GFK-CSCSGKDSVD---
15 Medicago_truncatula|ACJ83390|PKS1 VHRKS-L-LATL--GCK-CSCYDKNSVDVSD
16 Lotus_japonicus|Lj2g3v1280010|PKS1 AQRKS-F-LAGL--GCK-CYCSDKNSVDISD
17 Glycine_max|GLYMA_01G046600|PKS1 LQRKS-F-LAGL--GCK-CYCSDKNSVDISD
18 Glycine_max|GLYMA_02G106800|PKS1 LHRKS-F-LAGL--GCK-CYCSDKNSVDISD
19 Phaseolus_vulgaris|PHAVU_002G086000g|PKS1 LRRKS-F-LSSL--GCK-CYCSDSNSVDISD
20 Phaseolus_angularis|LR48_Vigan06g105400|PKS1 LRRKS-F-LASL--GCK-CYCSDSNSVDISD
21 Lupinus_angustifolius|TanjilG_14296|PKS1 VHAKS-F-LAGV--GLK-CSCSDKDSVDINS
22 Manihot_esculenta|cassava4.1_023142m|PKS1 KIGKN-F-LAVL--GCK-CSCSDKDSIDVDD
23 Manihot_esculenta|cassava4.1_006330m|PKS1 KIGKN-F-LADF-------------IDVDD
24 Manihot_esculenta|cassava4.1_022412m|PKS1 KIGKN-I-FAAL--GCK-CSCSDKDSIDVDD
25 Vitis_vinifera|CBI31966|PKS1 TPGKS-L-LASL--CCK-CSCSDKNSVETDE
26 Camellia_sinensis|XP_028087468.1|PKS1 RQGKS-L-LASL--GCN-CACNDKSSIDIDD
27 Theobroma_cacao|EOY01944|PKS1 VNGKT-F-LSGLV-ACK-CYCSDRDSVDIEE
28 Gossypium_arboreum|LOC108455995|PKS1 VNGKS-F-LSGLA-GCK-CYCSGRNSVEIEE
29 Gossypium_hirsutum|Gh_A03G0872|PKS1 VNGKS-F-LSGLA-GCK-CYCSGRNSVEIEE
30 Gossypium_hirsutum|Gh_D02G1256|PKS1 VNGKS-F-LSGLA-GCK-FYCSGRNSVDIEE
31 Gossypium_raimondii|B456_005G141100|PKS1 VNGKS-F-LSGLA-GCK-CYCSGRNSVDIEE
32 Gossypium_arboreum|LOC108466012|PKS1 ITAKS-F-LSCLP-ACK-CYCFDRSSIDIE-
33 Gossypium_hirsutum|Gh_A13G0651|PKS1 ITAKS-F-LSCLP-ACK-CYCFDRSSIDIE-
34 Gossypium_hirsutum|Gh_A13G0652|PKS1 ITAKS-F-LSCLP-ACK-CYCFDRSSIDIE-
35 Gossypium_hirsutum|Gh_D13G2533|PKS1 ITAKS-F-LSCLP-ACK-CYCFDRSSIDIE-
36 Gossypium_raimondii|B456_013G084800|PKS1 ITAKS-F-LSCLP-ACK-CYCFDRSSIDIE-
37 Corchorus_capsularis|CCACVL1_13934|PKS1 GNGKS-F-FSGLD-FCR-CYCSDRNSLDIEE
38 Prunus_persica|EMJ28355|PKS1 RQKKN-F-FASL--GCK-CSCSDKNSVDVDE
39 Fragaria_vesca_subsp._vesca|XP_004298433.1|PKS1 AQRKS-F-FAAL--G---CSCTDKNSVDINE
40 Arabis_alpina|AALP_AA5G017900|Brassicaceae_PKS1 SNKKS-F-LANF--GCK-CACSDENSVDVDE
41 Arabis_alpina|AALP_AA5G018000|Brassicaceae_PKS1 SNKKS-F-LATF--WCK-CACSDGNSVDVDE
42 Brassica_rapa_subsp._pekinensis|Bra024800|Brassicaceae_PKS1 NSKKS-F-ISNL--RCK-CVCTDENSVDVDD
43 Brassica_napus|BnaA06g33930D|Brassicaceae_PKS1 NSKKS-F-ISNL--RCK-CVCTDGNSVDVDD
44 Brassica_napus|BnaC07g22010D|Brassicaceae_PKS1 NSKKS-F-ISNL--RCK-CVCSDGNSVDVDD
45 Brassica_oleracea|Bo7g080840|Brassicaceae_PKS1 NSKKS-F-ISNL--RCK-CVCSDGNSVDVDD
46 Arabidopsis_lyrata|fgenesh2_kg.5__196__AT2G02950.1|Brassicaceae_PKS1 NNKKS-F-LANL--GCK-CACSDGNSVDVDD
47 Arabidopsis_thaliana|AT2G02950|Brassicaceae_PKS1 NNKKS-F-LANL--GCK-CACSDGDSVDVEE
48 Brassica_napus|BnaCnng39440D|PKS1 NNKKS-F-LSNL--RCK-C-CS-----DVDD
49 Brassica_oleracea|Bo2g136630|PKS1 NNKKS-F-LSNL--RCK-C-CS-----DVDD
50 Brassica_rapa_subsp._pekinensis|Bra026572|PKS1 NNKKS-F-LSNL--RCK-C-CS-----DVDD
51 Brassica_napus|BnaA02g26640D|PKS1 NNKKS-F-LSNL--RCK-C-CS-----DVDD
52 Brassica_rapa_subsp._pekinensis|Bra017406|PKS1 NNKKS-F-L-NL--GCK-CACSDGMSVDVDD
53 Brassica_napus|BnaA09g53700D|PKS1 NNKKS-F-LMNL--GCK-CACSDGISVDVDD
54 Brassica_napus|BnaC09g21140D|PKS1 NNKKS-F-LMNL--GCK-CACSDGISVDVDD
55 Brassica_oleracea|Bo9g072850|PKS1 NNKKS-F-LMNL--GCK-CACSDGISVDVDD
56 Helianthus_annuus|HannXRQ_Chr12g0380721|PKS1 SAVKS-L-FISL--G---CNCNDNKSVKITE
57 Cucumis_sativus|Csa_5G605030|PKS1 KNSSQ-L-AKDF--LYN-CYCWDNKSVESMN
58 Persea_americana|unknown_id|PKS1 MSGKN-F-FSNF--G---CNCASKKSVEVVE
59 Cucumis_sativus|Csa_2G108600|PKS1 NNNKS-F-LSNTLGYCM-CCTSNKKSTAVED
60 Helianthus_annuus|HannXRQ_Chr08g0210531|PKS1 SKSRT-F-LARF--G---CNCMNKKSTLIYE
61 Nymphaea_colorata|XP_031474915.1|PKS1 QKNRI-V-FMG-------CPCSGMKSVRTDD

B

```
                                                                          |....:.680|....:.690|....:.700|
 1 Musa_acuminata_subsp._malaccensis|GSMUA_AchrUn_randomG18930_001|PKS4 -AARR-F-F-----ARR-CPCSGGKSVDRES
 2 Persea_americana|unknown_id|PKS4                                     -SKRW-L-F-----GRK-CPCSGKKSVDVEE
 3 Nicotiana_attenuata|A4A49_03316|PKS4                                 -TRKW-F-F-----CRK-CPCSGKKSVQVEE
 4 Nicotiana_tabacum|LOC107796286|PKS4                                  -TRKW-F-F-----CRK-CPCSGKKSVQVEE
 5 Nicotiana_tabacum|LOC107784416|PKS4                                  -TRKW-F-F-----CRK-CPCSGKKSVQVEE
 6 Solanum_lycopersicum|Solyc10g081340.1|PKS4                           -TRKW-F-F-----CRK-CPCSGKKSVQVEE
 7 Solanum_tuberosum|PGSC0003DMG400028120|PKS4                          -TRKW-F-F-----CRK-CPCSGKKSVQVEE
 8 Medicago_truncatula|ABD28653|PKS4                                    -KPNW-F-L-----RRK-CPCTGKKSVQVNE
 9 Glycine_max|GLYMA_19G123300|PKS4                                     -KSIW-L-L-----RRK-CPCTGKKSVRVKE
10 Glycine_max|GLYMA_03G118900|PKS4                                     -KSIW-L-L-----RRK-CPCTGKKSVRVKE
11 Phaseolus_vulgaris|PHAVU_001G109100g|PKS4                            -KSSW-L-L-----RRK-CPCTGKKSVQIKE
12 Phaseolus_angularis|LR48_Vigan03g205600|PKS4                         -KSIW-L-L-----RRK-CPCTGKKSVQIKE
13 Manihot_esculenta|cassava4.1_025379m|PKS4                            -ATKW-L-L-----GRK-CPCSGKKSVQVEE
14 Populus_trichocarpa|POPTR_005G090000v3|PKS4                          -GTKW-L-L-----RRK-CPCSGKKSVQIEE
15 Vitis_vinifera|CBI31176|PKS4                                         -GRKW-F-F-----GRK-CPCSGKKAVEIQE
16 Theobroma_cacao|EOY09759|PKS4                                        -TIKW-L-W-----GRR-CPCSSKKSVQVEP
17 Gossypium_arboreum|LOC108459401|PKS4                                 -KLKW-L-W-----GRS-CPCSGNKSVQVEP
18 Gossypium_hirsutum|Gh_A05G3397|PKS4                                  -KLKW-L-W-----GRS-CPCSGNKSVQVEP
19 Gossypium_hirsutum|Gh_D04G0186|PKS4                                  -KLKW-L-W-----GRS-CPCSGNKSVQVEP
20 Gossypium_raimondii|B456_012G026500|PKS4                             -KLKW-L-W-----GRS-CPCSGNKSVQVEP
21 Gossypium_arboreum|LOC108462210|PKS4                                 -KLRR-L-W-----RRS-CPCSGKKSVQVEP
22 Gossypium_hirsutum|Gh_A13G0948|PKS4                                  -KLRR-L-W-----RRS-CPCSGKKSVQVEP
23 Gossypium_hirsutum|Gh_D13G1200|PKS4                                  -NLRR-L-W-----RLS-CPCSGKKSVQVEP
24 Gossypium_raimondii|B456_013G132100|PKS4                             -NLRR-L-W-----RLS-CPCSGKKSVQVEP
25 Corchorus_capsularis|CCACVL1_10672|PKS4                              IKCKW-L-W-----GPR-CPCSGKKSVQVDL
26 Corchorus_capsularis|CCACVL1_10673|PKS4                              IKCKW-L-W-----GPR-CPCSGKKSVQVDP
27 Daucus_carota_subsp._sativus|DCAR_009109|PKS4                        GTTKW-F-F-----RRK-CPCSGKKSVQVKE
28 Phalaenopsis_equestris|XP_020583039.1|PKS4                           -PGRR-F-F-----RRR-CPCSGKKSVDVEE
29 Cucumis_sativus|Csa_3G814370|PKS4                                    LAARW-I-FR----SSK-CPCTGKKSVQVQE
30 Camellia_sinensis|XP_028089884.1|PKS4                                -AKKW-F-L-----GRK-CPCSCKKSVEVKE
31 Fragaria_vesca_subsp._vesca|XP_011470920.1|PKS4                      PRVRW-FSF-----PRR-CPCSGKKSVRVEE
32 Amborella_trichopoda|AMTR_s00119p00112700|PKS4                       GASKW-S-F-----SCR-CPCYGKKSVGVRE
33 Chenopodium_quinoa|AUR62018475-RA|PKS4                               ISTKW-L-I----PRRK-CPCSGKKSVQVEE
34 Chenopodium_quinoa|AUR62020949-RA|PKS4                               ISTKW-L-I----PRRK-CPCSGKKSVQVEE
35 Beta_vulgaris_subsp._vulgaris|XP_019103906.1|PKS4                    ISTKW-L-I----PRRK-CPCSGKKSVQVEE
36 Arabis_alpina|AALP_AA8G035100|PKS4                                   -GSRW-F-F-----RRRTCPCSSSKSVQVKE
37 Arabidopsis_lyrata|scaffold_600360.1|PKS4                            -GPRW-F-F-----RRRTCPCSSSKSVQVQE
38 Arabidopsis_thaliana|AT5G04190|PKS4                                  -GPRW-F-F-----RRRACPCSSSKSVQVQE
39 Brassica_rapa_subsp._pekinensis|Bra009478|PKS4                       -GPRW-F-F-----RRRTCPCSSSKSVQVRE
40 Brassica_napus|BnaA10g26010D|PKS4                                    -GPRW-F-F-----RRRTCPCSSSKSVQVQE
41 Brassica_napus|BnaCnng10280D|PKS4                                    -GPRW-F-F-----RRRTCPCSSSKSVQVRE
42 Brassica_oleracea|Bo9g181340|PKS4                                    -GPRW-F-F-----RRRTCPCSSSKSVQVRE
43 Brassica_napus|BnaA01g07730D|PKS4                                    -GPRW-F-F-----RRRTCPCSSSKSVQVQE
44 Brassica_oleracea|Bo2g005470|PKS4                                    -GPRW-F-F-----RRRTCPCSSSKSVQVQE
45 Brassica_napus|BnaAnng17720D|PKS4                                    -GSRW-F-F-----RRRTCPCSSSKSVQVQE
46 Nymphaea_colorata|XP_031479521.1|PKS4                                GSGRW-R-F-----GRQ-CPCICKKSIAVEE
47 Oryza_brachyantha|OB01G20500|PKS4                                    -GQRG-GLF-----SRD-CPCAGRKAVTVDV
48 Oryza_glaberrima|ORGLA01G0093300|PKS4                                -GQRW-GLF-----SRD-CPCAGRKAVTVDV
49 Oryza_nivara|ONIVA01G12840|PKS4                                      -GQRW-GLF-----SRD-CPCAGRKAVTVDV
50 Oryza_sativa_subsp._japonica|BAA90343|PKS4                           -GQRW-GLF-----SRD-CPCAGRKAVTVDV
51 Oryza_rufipogon|ORUFI01G11300|PKS4                                   ------------------EHCAGRKAVTVDV
52 Oryza_punctata|OPUNC01G10250|PKS4                                    -GQRW-RLF-----SRD-CPCAGRKAVTVDV
53 Hordeum_vulgare_subsp._vulgare|HORVU3Hr1G037030|PKS4                 -GQRW-GIF-----SRD-CPCAGRKAVTVDV
54 Triticum_aestivum|TraesCS1B01G185300|PKS4                            -GQRW-GIF-----SRD-CPCAGRKAVTVDV
55 Triticum_aestivum|TraesCS3A01G181000|PKS4                            -GQRW-GIF-----SRD-CPCAGRKAVTVDV
56 Triticum_aestivum|TraesCS3B01G211100|PKS4                            -GQRW-GIF-----SRD-CPCAGRKAVTVDV
57 Sorghum_bicolor|SORBI_3003G121700|PKS4                               -GQRW-TLF-----GRD-CPCAGRKAVTVDV
58 Zea_mays|Zm00001d008296|PKS4                                         -AQRW-TLF-----GRD-CPCAGRKAVTVGI
59 Setaria_italica|XP_004971565.1|PKS4                                  -GQRW-TLF-----GRD-CPCAGRKAVTVDV
60 Prunus_persica|EMJ06068|PKS4                                         LGVRW-YSIFPMRRSRR-CPCFGKKSVRVVD
```

Supplemental Figure S3. Alignments of Motif C of PKS1 and PKS4 proteins. Supports Figure 2.

(A) Alignment of motif C from PKS1 homologs. Cys residues are indicated in yellow. The most highly conserved Cys residue, called Cys-12 in the manuscript, is indicated with an arrow. **(B)** Alignment of motif C from PKS4 homologs. Cys residues are indicated in yellow. The most highly conserved Cys residue, called Cys-12 in the manuscript is indicated with an arrow.

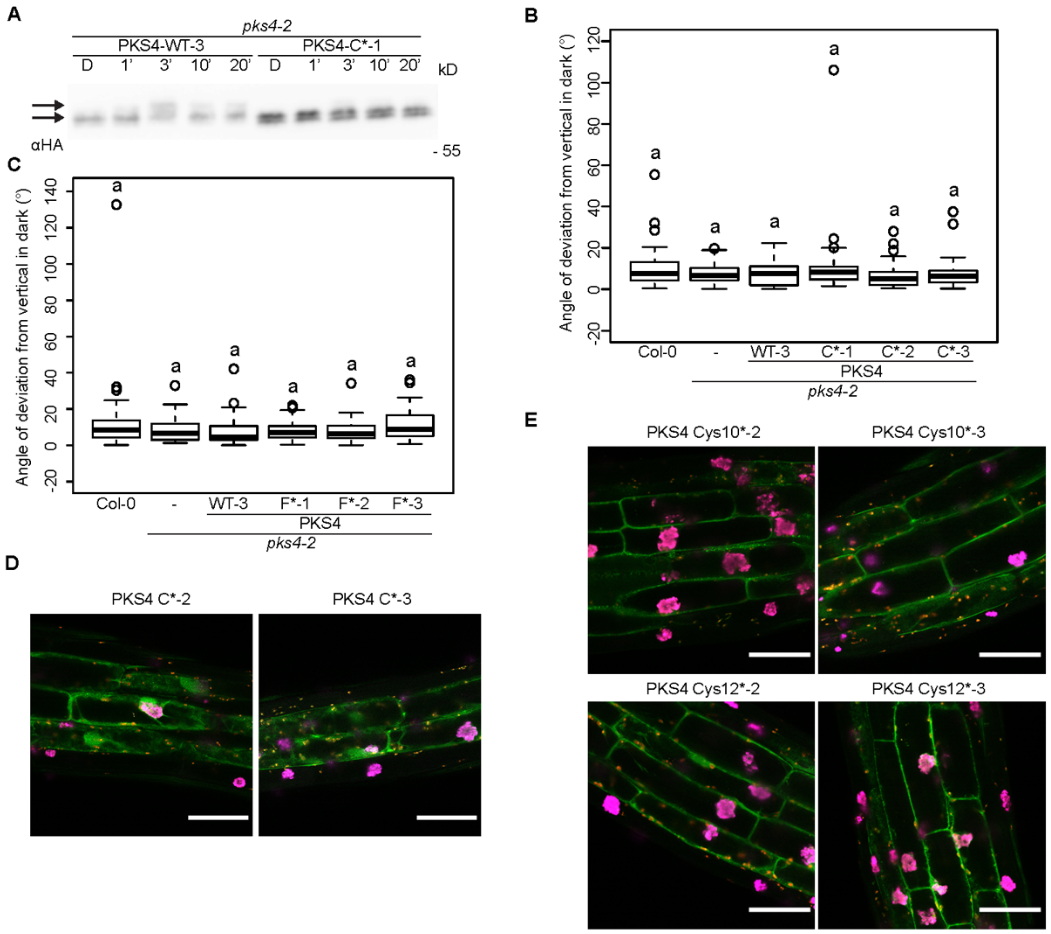

Supplemental Figure S4. Further characterization of motif C and motif F mutants (C* and F*). Supports Figures 6, 7 and 8.

(A) Immunoblot analysis with anti-HA antibody of samples extracted from *pks4-2 PKS4* WT and *pks4-2 PKS4 C**-1 3-d-old dark grown seedlings exposed to 1 µmol m^{-2} s^{-1} blue light for 1, 3, 10, or 20 min. This immunoblot was obtained using samples from a different line than the one shown in Figure 6C. **(B)** Hypocotyl growth orientation of 3-d-old Col-0, *pks4-2*, *pks4-2 PKS4* WT-3 and *pks4-2 PKS4 C**-1, *C**-2, and *C**-3 etiolated seedlings. 0° represents vertical growth. We consider the absolute value of the angle, whether the seedling bends towards the left or the right side. n= 50 – 60, different lowercase letters are significantly different (P > 0.01, one-way ANOVA with Tukey's HSD test). **(C)** Hypocotyl growth orientation of 3-d-old Col-0, *pks4-2*, *pks4-2 PKS4* WT-3 and *pks4-2 PKS4 F**-1, *F**-2, and *F**-3 etiolated seedlings. Considerations and data analysis were as in panel B. **(D)** Confocal microscopy images of 3-d-old *pks4-2* dark-grown seedlings harboring *proPKS4:PKS4 C*:GFP* in cortex cells. These images were obtained using different lines from those presented in figure 7C. Scale bars, 50 µm. **(E)** Confocal microscopy images of 3-d-old *pks4-2* dark-grown seedlings carrying *proPKS4:PKS4 Cys10*-GFP* or *proPKS4:PKS4 Cys12*-GFP* in cortex cells. These images were obtained using different lines from those presented in figure 7D. Scale bars, 50 µm. Note that these lines also show oil bodies (in magenta) resulting from the expression of *OLE1-RFP* from the *OLE1* promoter, used as a seed coat selection marker.

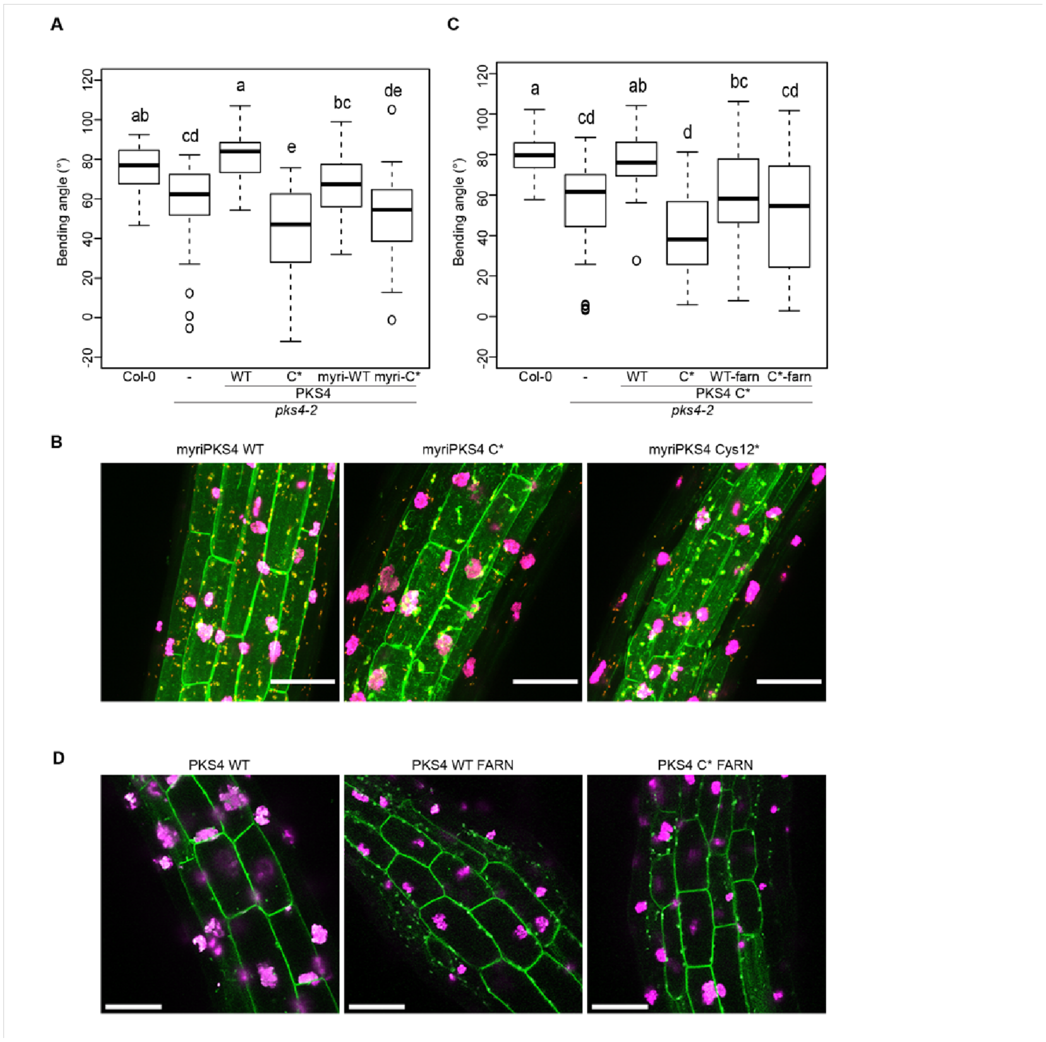

Supplemental Figure S5. Targeting PKS4 C* to the PM through farnesylation does not rescue PKS4 function. Supports Figure 9.

(A) Phototropic curvature of 3-d-old dark-grown Col-0, *pks4-2*, *pks4-2 PKS4* WT-3, *pks4-2 PKS4 C**-2, *pks4-2 myriPKS4* WT and *pks4-2 myriPKS4* C* seedlings treated with unidirectional blue light. Primary transformants in the *pks4*-2 background expressing *PKS4* WT and the different variants were assayed. Seedlings were exposed to 0.1 µmol m^{-2} s^{-1} blue light during 24 h prior to measurement of growth reorientation. n= 40 – 60, different lowercase letters are significantly different (P > 0.01, one-way ANOVA with Tukey's HSD test). **(B)** Confocal microscopy images of the same samples shown in Figure 9E but as a maximal projection of a cortex cell. Note the larger intracellular structures particularly in lines *myriPKS4 C** and *myriPKS4 Cys-12**, which correspond to stromules (see Figure 9F). **(C)** Phototropic curvature of 3-d-old dark-grown Col-0, *pks4-2*, *pks4-2 PKS4 C**-2, *pks4-2 PKS4* WTfarn and *pks4-2 myriPKS4 C*farn* seedlings treated with unidirectional blue light. Considerations, light treatment, and data analysis were as in S3A. **(D)** Confocal microscopy images of 3-d-old etiolated hypocotyls cortex cells expressing *PKS4-GFP*, *PKS4-GFPfarn*, and *PKS4 C*-GFPfarn* (green signal) from the *PKS4* promoter. Note that these lines also show remaining oily bodies (in magenta) resulting from the expression of *OLE1-RFP* from the *OLE1* promoter, used as a seed coat selection marker. Scale bars, 50 µm.

Supplemental Table S1. Statistical data

Figure	Variable	Parameter (alpha)	Degree of freedom	Test statistics (F value)	
Figure 6B	Genotype	0.01	5	44.89	
Figure 6D	Genotype	0.01	5	42.51	
Figure 7E	Genotype	0.01	5	35.23	
Figure 8C	Genotype	0.01	5	27.84	
Figure 8D	Genotype	0.01	5	15.97	
Figure 9B	Genotype	0.01	5	17.02	
Figure 9C	Genotype	0.01	5	14.15	
Figure 9D	Genotype	0.01	4	36.68	
Figure S3B	Genotype	0.01	5	2.358	
Figure S3C	Genotype	0.01	5	2.65	
Figure S4A	Genotype	0.01	5	33.59	
Figure S4C	Genotype	0.01	5	16.82	

Supplemental Table S2. Primers used in this study

Name	Sequence
CF129	5'- ggggtaccaaaatggtgacactaacacca-3'
CF470	5'- cgcggatccccttttccttgaaggaactgttg-3'
CF471	5'- cgcggatccctgtgtcgtcctcctctgttc-3'
CF472	5'- cggggtaccaaaatgaagagtgaagggagtgattc-3'
CF473	5'- cgcggatcccctgactataaagaagagatg-3'
CF507	5'- cggggtaccaaaatgaagaacagtaatggtcaga-3'
