# Supplementary material for: Protein S-acylation controls the subcellular localization and biological activity of PHYTOCHROME KINASE SUBSTRATE
Source: Plant Cell. 2023 Mar 28;35(7):2635–53. doi: 10.1093/plcell/koad096 (PMC10291038; doi:10.1093/plcell/koad096)
Supplement: koad096_Supplementary_Data [file tpc.22.00662Peer Review Report.pdf › tpc.22.00662Peer Review Report.pdf]

Protein S-acylation controls the subcellular localization and biological activity of PHYTOCHROME KINASE SUBSTRATE

Ana Lopez Vazquez, Laure Allenbach Petrolati, Martina Legris, Christophe Dessimoz,
Edwin R. Lampugnani, Natasha Glover and Christian Fankhauser

Corresponding author: name email (hristian.fankhauser@unil.ch/9)

Review timeline:

**TPC2022-RA-00662**	Submission received:	June 11, 2022
	1st Decision:	September 2, 2022 *revision requested*
**TPC2022-RA-00662R1**	1st Revision received:	December 20, 2022
	2nd Decision:	February 14, 2023 *accept with minor revision*
**TPC2022-RA-00662R2**	2nd Revision received:	February 24, 2023
	3rd Decision:	Mar. 4, 2023 *acceptance pending, sent to science editor*
	Final acceptance:	Mar. 22, 2023

REPORT: (The report shows the major requests for revision and author responses. Minor comments for revision and miscellaneous correspondence are not included. The original format may not be reflected in this compilation, but the reviewer comments and author responses are not edited, except to correct minor typographical or spelling errors that could be a source of ambiguity.)

TPC2022-RA-00662 1st Editorial decision – *revision requested* September 2, 2022

We have received reviews of your manuscript entitled "Control of PHYTOCHROME KINASE SUBSTRATE subcellular localization and biological activity by protein S-acylation." Thank you for submitting your best work to The Plant Cell. The editorial board agrees that the work you describe is substantive, falls within the scope of the journal, and may become acceptable for publication, pending revision and potential re-review. However I should stress that we are reluctant to see manuscripts undergoing multiple rounds of revision and would be unlikely to offer you more than one chance to satisfy the reviewers.

Reviewer #2 in particular raised concerns that may require additional experiments to address, please pay special attention to these requests.

Reviewer #1 made this statement in their review: "Neither Jpred-4 nor AlphaFold would be an appropriate tool to predict intrinsically disordered regions. It might be safer just to say that these algorisms failed to predict the structure." I disagree with this statement; however, there are better predictors in my opinion, including D2P2, which is a consensus disorder predictor, and Metapredict. You may choose to include data from these disorder predictors to support your statements on predicted disorder. People in the IDR field would prefer NMR data to support that these are disordered regions; however, stating these are predicted to be disordered is fine.

-- Reviewer comments:

TPC2022-RA-00662R1 1st Revision received December 20, 2022

Reviewer comments and **author responses**:

Reviewer #1:

This manuscript describes how PKS proteins localize to the plasma membrane. PKSs are quite interesting and important protein, which is involved both in phototropin- and phytochrome-mediated responses. Although they have been known quite a while, the mechanisms of their action remain obscure. The authors demonstrate that membrane

localization of PKSs is facilitated by a certain motif within the molecule through S-acylation. They further demonstrate the importance of the localization for the biological activity. The work provides a significant step toward understanding how PKS mechanistically functions at the molecular level. The experiments are designed well, and results are mostly clear.

RESULTS (line 119-): Neither Jpred-4 nor AlphaFold would be an appropriate tool to predict intrinsically disordered regions. It might be safer just to say that these algorisms failed to predict the structure.

As suggested by the editor we used the D2P2 predictor for PKS1 to PKS4 (Arabidopsis sequences) and present the output as Supplementary Figure 1. This predictor which takes into consideration a variety of prediction methods suggests that PKS1-PKS4 are largely intrinsically disordered and contain no domains of known function. We changed the text accordingly.

RESULTS (Fig 3, Fig 5, Fig S2, Fig S3): I agree that PKS4 C* most likely localizes in the cytosol. However, its cytosolic localization shown by GFP fluorescence is less clear than those of PKS1 derivatives (Fig 2, Fig 5). If there is a particular reason for that, please explain.

This is a good comment, we did not explain this carefully enough. In the first 2 figures presenting microscopy data we show seedlings expressing GFP fused PKS1 or PKS4 variants driven by the 35S promoter. This leads to high expression, and we performed microscopy in epidermal hypocotyl cells which are the easiest to visualize. In the subsequent figures we imaged *pks4* transformed with *PKS4pro:PKS4-GFP* (and variants of this constructs). Such seedlings express significantly lower levels and microscopy was performed in hypocotyl cortex cells because we found that PKS4 is more expressed in the cortex than the epidermis.

These experiments are more challenging because the signal is lower and we image deeper in the tissue. This is now clearly explained in the text. Moreover, we provide new microscopy data (more lines and better images) presented in figures 7C, 7D, 8E, 8F, S3D, S3E and S4B.

RESULTS (line 203-, Fig 3); METHODS (line 559-): It might be better to briefly explain how the hydroxylamine functions in the assay

We now detail the principle of this assay in the Methods section.

Text change. DISCUSSION (line 302-). As agreed by the authors, the myristoylation sequence partially complemented the phototropic phenotype caused by the C* mutation (line 290-, Fig 5B). In my opinion, this supports the authors' hypothesis that plasma membrane localization is important for the biological activity of PKSs. The authors might refer to this aspect.

We stayed cautious in the discussion for 2 reasons. First, myristoylation does not perfectly restore the subcellular localization of PKS4 C* mutants (see Figures 9 and S4). In addition, while there is a tendency for myri-C* mutant lines to better complement than C* mutant lines, neither of these constructs actually complement *pks4*.

DISCUSSION (line 412-). The authors state that PKSs form a complex with phot and NPH3 (line 105-, line 176-). I wonder whether the NPH3 cycling is affected in the pks4 mutation and PKS 4C* mutation. I understand that such experiments are beyond the scope of this work but would like to know the authors' opinion about such an experiment.

This is an excellent question, and we would love to know the answer. Unfortunately, we currently do not have the lines to answer your question.

<u>Reviewer #2:</u>

This is an interesting paper on further understanding how PKS proteins function in tropic responses; the manuscript is well written and their figures are of a high quality, especially the microscopy. However, I feel that more work is needed to really understand the importance of the cysteine residue(s) described in this manuscript before publication can be considered. I am surprised the authors did not investigate the protein complexes PKS forms in the presence and absence of these cysteine mutations as this would strongly strengthen their work. Without these additional experiments, I feel that their conclusions are very limited and is hence not suitable for publication in Plant Cell currently.

In figure 5, the authors demonstrate that a myri-tag is sufficient to restore PKS4 C* localisation to the PM but it cannot rescue the PKS4 C* phenotype. The myri-tagged WT PKS4 (in 2 of the 3 independent lines) does complement the pks4 mutant, suggesting that the myri tag isn't directly interfering with PKS4 activity. Moreover, in Figure 4B/D there is a strong phenotypic effect of the C* mutations on PKS4 functional activity but only a very small change in the localisation of PKS4 C* (Sup Fig 2F). Taking these points together, it's unclear to me if the changes in localisation can properly explain the phenotype of PKS4 C*. As mentioned above, further work is needed to characterise the protein complexes of PKS4 C* compared to WT PKS4.

We thank the reviewer for these comments. As indicated by the reviewer adding lipid modification tags to PKS4 C* mutants either by N-terminal myristoylation or C-terminal farnesylation was insufficient to provide complementation potential to these PKS4 variants (now presented in Figures 9B, 9D, S4A and S4C). However, the addition of these tags tended to improve phototropism compared to C* mutants without tags. In contrast, when these tags were added to PKS4 WT then they rather diminished the potential of PKS4 to complement the mutant (9C, S4C). Furthermore, closer microscopic examination showed that while the myri-tag brings PKS4 C* variants to the cellular periphery (Figures 9E, F, S4B and S4D) it also led to localization of these proteins to structures known as stromules (Figure 9F, S4). We therefore discuss this data more cautiously in the revised manuscript. We conclude that these tags do not perfectly rescue the subcellular localization of the C* mutants and do not allow to restore their function for phototropism. Potential hypotheses explaining these finding are presented at the end of the discussion.

To address the last point of your comment, we did cell fractionation experiments and co-IP using protein extracts from etiolated seedlings expressing *PKS4p:PKS4-HA* (WT or C* mutant). This experiment showed that both the WT and C* mutant are in microsome fractions (Figure 7A). Hence the PKS4 C* is not cytosolic but associated with a membrane compartment (e.g. ER). We have not characterized this further. However, solubilized microsomes where immunoprecipitated and we found that NPH3 associated with both WT and the C* PKS4 mutant (Figure 7B).

In this revised manuscript we also show complementation data for two additional PKS4 motif C mutants. The initial C* mutant has two Cys mutated to Ser (Cys10 which is not present in all PKS proteins and the invariant Cys12). Our new data shows that Cys12 but not Cys10 is required for phototropic function and for plasma-membrane association (Figures 9D and E). Collectively we find a very strong correlation between the propensity of PKS4 variants to localize to the plasma-membrane and their function in phototropism. The PKS4 C* mutant was still able to interact with NPH3 and was likely phosphorylated by phot1 in response to blue light (light-induced mobility shift, Figures 6C and S3A) although less than the WT, which is not unexpected given its altered localization. However, we agree that adding lipid tags favoring plasma membrane localization was not sufficient to restore the function of these variants. One possibility is that motif C allows plasma-membrane association though the middle of the protein, while the tags either tether the protein to the plasma membrane though the N- or the C-terminus.

I also have concerns over potential degradation / cleavage of their PKS4 C* 2-line (Figure 4C). In their westerns (4C), there are 3 mutant bands on the gel, 2 of which are running below the non-phosphorylated WT band. The explanation the authors provide in text is not sufficient to explain these observations as in 4A (where protein samples are from the dark) the same protein is now running at the same size as WT. Therefore, it cannot be an effect of the mutation or post-translational modification as the authors suggest in text. Instead, it seems light dependent. Do the other two transgenic lines described in 4A behave in a similar manner in the light as C*-2?

We thank the reviewer for carefully looking at the data. The gels used for former figures 4A and 4C (now 6A and 6C) are not exactly the same because they serve different purposes. For 6A our aim was to find lines with similar protein levels of WT and C* mutants, hence we did not try to resolve different isoforms. In contrast for 6C, we wanted to detect the light- induced mobility shift of PKS4, which requires a different way of running the gel to resolve isoforms migating slightly differently. By running those gels, we also noticed that in etiolated seedlings the C* mutant migrates as 2 isoforms (samples marked D in Fig. 6C and S3A). To ensure that this is not an artifact of a particular line, we made the same experiment with another C* mutant line. This data is now presented on Figure S3A. In the Methods section we now explain how the gels presented in Figures 6C and S3A differ from the others.

The authors investigate the localisation of two different cell types: Fig 2 / 3 are epidermal cells in the hypocotyl hook. Fig 5, Sup fig 3 (maybe Sup fig 2?) are from the hypocotyl cortex. Is there a reason why the authors switch between different cell types, and do they think this could influence the differences in phenotypic effect of the PKS4 C* mutations between Fig 3 (strong effect) and Fig 5 / Sup fig 2 / Sup fig 3 (weak effect)?

We apologize for not explaining this carefully enough. Please also check our answer to reviewer 1. The first 2 figures showing microscopy images (now figures 4 and 5) were done with plants expressing GFP tagged PKS proteins driven by the *35S* promoter. This facilitates microscopic examination for 2 reasons. First, the lines express more proteins and second it allows microscopic examination in the epidermis. All subsequent microscopic images were done with GFP-tagged PKS4 (WT and various mutants) driven by the *PKS4* promoter, which allows the examination of the tagged protein expressed at physiological levels. This experiment showed that PKS4 (as phot1) is more strongly expressed in cortex cells, which is why we examined PKS4-GFP in these cells. However, the microscopy is more challenging due to lower expression and observations deeper in the tissue. We now present new and more detailed microscopy data, see figures 6C, 6D, 7E, 7F, S3D, S3E and S4B.

Other concerns Minor concerns:

What does PKS-like mean? Similarly, what does PKS1-like mean?

This term is ambiguous and we removed it from the manuscript. We now define PKS proteins as proteins that contain the 6 motifs A to F.

Check text and specify. Regarding the conserved motifs: Position of the motifs needs to be stated on the figure in 2A and somewhere in the manuscript.

This information is now presented on revised Figure 4A (former figure 2A)

What is the average length and degree of conservation of these residues amongst PKS proteins e.g. in Arabidopsis?

The conservation of these sequence motifs amongst different PKS proteins is represented in the different LOGOs (the larger the letter the higher the degree of conservation). We don't think that presenting % of conservation specifically amongst Arabidopsis PKS proteins is particularly relevant.

I think the authors should state where the phot1 phosphorylation site is in relation to these motifs.

The light induced phosphorylation site of PKS4 described in Schumacher et al., 2018 (Ser299) is between motifs D and E. This is now indicated in the discussion

The authors need to better describe what they mean by C and F are related.

We reorganized the figures to make this more easily apparent (see Figure 2B). Moreover, we have shown that this relatedness is significant (see methods section).

As above, the authors also need to better describe what they mean by BIG GRAIN and D are related.

Thanks for asking us to look at this in more detail. The new experiment we performed showed that the homology between PKS and BIG GRAIN (BG) proteins is actually more extended than we initially found. To address this question, we collected BG sequences from OMA orthology database (https://omabrowser.org/oma/home/), aligned all PKS and BG protein sequences and looked for conserved motifs. This analysis showed that PKS and BG proteins share 2 motifs (D and A). This information is presented in new Figure 3.

Please state whether the imaging was repeated and the approximate number of samples analysed for each of the different conditions. If the authors are using different tissue types, I think it would also be necessary to clearly state in the methods which cell type is being imaged for each figure.

All microscopy experiments were performed multiple times with more than one transgenic line. As PKS4 C* is particularly relevant we have shown data for multiple lines in the paper (Figure 7C, 7D, S3S and S3E). We now indicate when hypocotyl epidermal cells are imaged (Figures 4 and 5) and when we image hypocotyl cortex cells (*PKS4* promoter driven constructs on Figures 7, 8, 9, S3 and S4).

TPC2022-RA-00662R1 2nd Editorial decision – *accept with minor revision* February 14, 2023

We have received reviews of your manuscript entitled "Control of PHYTOCHROME KINASE SUBSTRATE subcellular localization and biological activity by protein S-acylation." On the basis of the advice received, the board of reviewing editors would like to accept your manuscript for publication in The Plant Cell.

This acceptance is contingent on revision based on the comments of our reviewers. In particular, Reviewer #2 noted several conclusions for which you may want to consider softening the language. Reviewer #2 also suggests that additional experiments are needed to support some of your conclusions, but I do not agree; however, you may want to consider whether you would like to alter the language around these points.

This being said, as editor, I am satisfied with the current version of the manuscript and am using this revision process as a mechanism for you to consider these new comments/suggestions and make edits in response as you like.

TPC2022-RA-00662R2 2nd Revision received February 24, 2023

Reviewer comments and **author responses**:

Reviewer #1:

I am satisfied with the revision. The authors did a good job.

We thank reviewer 1 for evaluating our work.

Reviewer #2:

Overall, the manuscript is much improved, and I thank the authors for their extensive further experimentations and changes to the manuscript. The data on PKS4 localising to microsomal fractions and being able to associate with NPH3 within these fractions is highly interesting and worth further studies. My remaining concerns are described below.

We thank reviewer 2 for evaluating our work.

Main concerns

I think the language used surrounding the conclusion is overly strong and needs either a re- think or further experiments. For example:

L48 - "Our work identifies the mode of plasma membrane association of PKS proteins"

This phrase was changed to present our data more cautiously.

L271 - "PKS4 C* revealed that it does not localise to the plasma membrane".

This phrase was changed to present our data more cautiously.

L298/299 - "very strong correlation between the ability of PKS4 to associate with the plasma membrane"

This phrase was changed to present our data more cautiously.

L318/319 - "highly conserved Cys residues in motif is essential for the... plasma membrane localisation of the protein".

This phrase was changed to present our data more cautiously.

TPC2022-RA-00662R2 3rd Editorial decision – *acceptance pending* March 4, 2023

We are pleased to inform you that your paper entitled "Control of PHYTOCHROME KINASE SUBSTRATE subcellular localization and biological activity by protein S-acylation" has been accepted for publication in The Plant Cell, pending a final editorial review by a science editor.

Final acceptance from Science Editor Mar. 22, 2023
